# A reference genome of the European beech (*Fagus sylvatica* L.)

**DOI:** 10.1093/gigascience/giy063

**Published:** 2018-05-28

**Authors:** Bagdevi Mishra, Deepak K Gupta, Markus Pfenninger, Thomas Hickler, Ewald Langer, Bora Nam, Juraj Paule, Rahul Sharma, Bartosz Ulaszewski, Joanna Warmbier, Jaroslaw Burczyk, Marco Thines

**Affiliations:** 1 Senckenberg Biodiversity and Climate Research Centre (BiK-F), Senckenberg Gesellschaft für Naturforschung, Senckenberganlage 25, D-60325 Frankfurt am Main, Germany; 2 Goethe University, Department for Biological Sciences, Institute of Ecology, Evolution and Diversity, Max-von-Laue-Str. 9, D-60438 Frankfurt am Main, Germany; 3 Johannes Gutenberg Universität, Fachbereich Biologie, Institut für Organismische und Molekulare Evolutionsbiologie (iOME), Gresemundweg 2, 55128 Mainz; 4 Goethe University, Department for Geology, Institute of Geography, Max-von-Laue-Str. 23, D-60438 Frankfurt am Main, Germany; 5 University of Kassel, FB 10, Department of Ecology, Heinrich-Plett-Str. 40, D-34132 Kassel, Germany; 6 Senckenberg Research Institute and Natural History Museum Frankfurt, Department of Botany and Molecular Evolution, Senckenberg Gesellschaft für Naturforschung, Senckenberganlage 25, D-60325 Frankfurt am Main, Germany; 7 Kazimierz Wielki University, Department of Genetics, ul. Chodkiewicza 30, 85-064 Bydgoszcz, Poland

**Keywords:** *forest tree*, *fungi*, *genomics*, *hardwood*, *hybrid assembly*, *transcriptomics*

## Abstract

**Background:**

The European beech is arguably the most important climax broad-leaved tree species in Central Europe, widely planted for its valuable wood. Here, we report the 542 Mb draft genome sequence of an up to 300-year-old individual (Bhaga) from an undisturbed stand in the Kellerwald-Edersee National Park in central Germany.

**Findings:**

Using a hybrid assembly approach, Illumina reads with short- and long-insert libraries, coupled with long Pacific Biosciences reads, we obtained an assembled genome size of 542 Mb, in line with flow cytometric genome size estimation. The largest scaffold was of 1.15 Mb, the N50 length was 145 kb, and the L50 count was 983. The assembly contained 0.12% of Ns. A Benchmarking with Universal Single-Copy Orthologs (BUSCO) analysis retrieved 94% complete BUSCO genes, well in the range of other high-quality draft genomes of trees. A total of 62,012 protein-coding genes were predicted, assisted by transcriptome sequencing. In addition, we are reporting an efficient method for extracting high-molecular-weight DNA from dormant buds, by which contamination by environmental bacteria and fungi was kept at a minimum.

**Conclusions:**

The assembled genome will be a valuable resource and reference for future population genomics studies on the evolution and past climate change adaptation of beech and will be helpful for identifying genes, e.g., involved in drought tolerance, in order to select and breed individuals to adapt forestry to climate change in Europe. A continuously updated genome browser and download page can be accessed from beechgenome.net, which will include future genome versions of the reference individual Bhaga, as new sequencing approaches develop.

## Data Description

### Context

European beech (*Fagus sylvatica* L., NCBI Taxon ID: 28 930) is one of the most important and widespread broad-leaved tree species in Europe. Its natural range extends from southern Italy to southern Scandinavia and from the Iberian Peninsula to Crimea [1]. Under favourable conditions, in particular in Central Europe, it can outcompete all other tree species and form monospecific stands in which, due to shading, other broad-leaved species can hardly establish [2]. Because of their cultural and environmental importance, as well as their global uniqueness, ancient and primeval beech forests in Europe, with five areas located in Germany, have been listed as UNESCO World Heritage sites [3]. Langer et al. [4] analyzed the species composition of these forests and concluded a need for conservation of near natural or primeval beech forest stages for their richness in fungal species.

There have been 1,766 fungal species reported associated with beech, ranging from general commensals to specialised pathogens and symbionts, such as the very common obligate mycorrhizal symbiont *Lactarius blennius* (beech milkcap), with a distribution corresponding to the natural distribution of beech [5, 6]. On average, 25 fungal species are associated with the dead wood of *F. sylvatica* [7]. Among them are threatened species and species with natural value such as *Hericium coralloides* and *Phleogena faginea* [8, 9]. Nitrogen uptake by beech roots is also highly dependent on the mycorrhizal community [10]. Thus, the European beech is in intimate contact with a variety of fungi.

Even though its natural area of dominance [11] has been reduced by land use and planting other commercially important species, such as Norway spruce (*Picea abies*; [12]), European beech remains an important hardwood species on the European scale. However, as European beech does not cope very well with dry and hot conditions, fire, and flooding, its suitability under a potentially more extreme climate in the future is debatable [13]. Thus, genetic and genomic data are crucial for understanding its adaptive capacity, in particular, under climate change [14], which will also lead to a change in biotic stress, including fungal pathogens [15, 16].

Several tree genomes have been released over the past decade, among them oaks [17, 18] and Chinese chestnut [19] of the beech family (*Fagaceae*). However, despite its economic and ecological importance, genetic and genomic resources in the genus *Fagus* (beeches) are limited to some studies on the genetic diversity and candidate genes using single-nucleotide polymorphism data [20-23], a few genome-wide associations studies [24, 25], investigation into methylation patterns [26], and some transcriptome data [27, 28]. Thus, it was our aim in this study to provide a draft assembly of the European beech and to make it available to the research community for in-depth analyses and follow-up studies, taking advantage of the genomic resource. The risk of contamination with a variety of microorganisms, including bacteria and the numerous fungi found in association with trees in general and beech in particular [29], is high when conducting sampling of specimens from nature, as evidenced by the large amount of contaminant DNA in the effort of sequencing the olive tree genome from a 1,000-year-old individual [30]. Thus, we are also describing a method of DNA extraction from dormant buds that, in our case, led to the absence of contaminant organisms in the assembly.

### Methods

#### Selection of the sequenced individual

**Figure 1:**
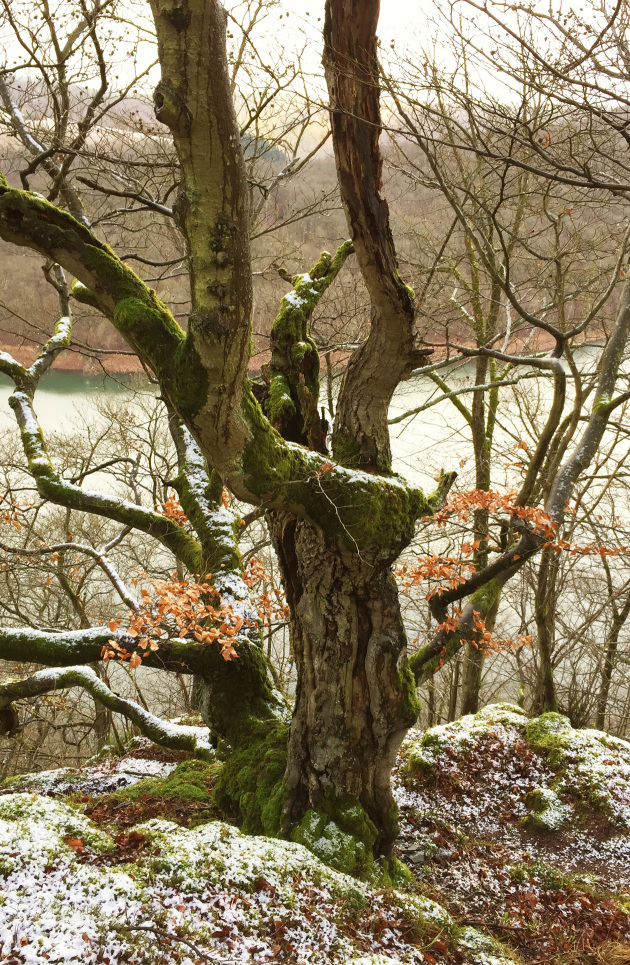
The sequenced individual Bhaga at the time of sampling. Note the very low branching on the cliff, with a major part of the individual reaching over the edge.

For the genome sequencing, an individual tree standing on a rocky outcrop on the rim of a scarp to the Edersee (German Kellerwald-Edersee National Park) was selected (Fig. 1). The individual, named Bhaga (the reconstructed common root of the common name of the tree in several European languages), is estimated to be up to 300 years old, based on its poor stand, low branching, as well as bark and stem characteristics. A direct measurement was not possible because the trunk is not fully preserved due to the tree's age. An old individual was selected to avoid the influence of modern forestry on the tree's genetic makeup .

#### Flow cytometric genome size and nucleotide composition

Relative genome size and absolute genome size was estimated by flow cytometric analyses of fresh leaf buds using a CyFlow space (Partec, Münster, Germany). Leaf buds (without bud scales) of the analysed sample and leafs of the internal standard (Glycine max cv. “Polanka” (2C = 2.50 pg) were treated and analysed as described previously [31].

#### DNA and RNA extraction

A modified protocol based on the standard CTAB (cetyl trimethylammonium bromide) method described by Doyle and Doyle [32] was applied. The CTAB extraction buffer consisted of 100 mM Tris-HCl, 20 mM Ethylenediaminetetraacetic acid (EDTA), 1.4 M NaCl, 2% CTAB, 0.2% *ß*-mercaptoethanol, and 2.5% polyvinylpyrrolidone. For DNA extractions, about 100 buds (collected in February 2015) with a few millimeters of the subtending branchlets were cut from twigs of a larger branch, transported to the laboratory on ice, and surface sterilized by gentle shaking for 2 minutes in 4% sodium hypochlorite solution containing 0.1% Tween. Subsequently, the buds were rinsed with sterile water until no foam formation was evident. Then, the water was poured off and the buds were descaled after cutting off the subtending branchlet with sterile scalpels. The dormant leaf tissue in the buds was ground in liquid nitrogen using a mortar and pestle. A total of 1,200 mg of powdered tissue was distributed to 24 2-mL reaction tubes. Each sample was thoroughly mixed with three 3-mm metal beads in 600 µL of extraction buffer and incubated at 60°C for 30 minutes. After this, 600 µL of phenol:chloroform:isoamyl alcohol (25:24:1) (PCI) was added, and the tubes were gently mixed by inversion. Subsequently, the tubes were centrifuged at 19,000 *g* for 2 minutes. Next, 500 µL of the supernatant were transferred to a new tube and 600 µL of PCI was added. The tubes were centrifuged again for 2 minutes, and each 500 µL of the supernatant transferred to a new tube. Subsequently, 15 µL RNase A solution (100 mg/mL) were added to each tube, and the tubes were incubated at 37°C for 30 minutes. After the incubation, 600 µL of chloroform was added, and the tubes were gently shaken. Subsequently, the tubes were centrifuged at 19,000 *g* for 2 minutes. The supernatant of all tubes was transferred to a 45-mL reaction tube. Then, 3 M sodium acetate solution at pH 5.3 (supernatant to 3 M sodium acetate solution = 1:0.09) and 100% ethanol (supernatant to ethanol = 1:2) were added to the supernatant, and the tube was gently mixed by inversion. Afterward, it was incubated at –20°C for 30 minutes and centrifuged at 4800 *g* for 3 minutes at 4°C. The supernatant was carefully poured off, and the pellet was washed twice with 70% ethanol. After a final centrifugation at 4800 *g* for 2 minutes at 4°C, the supernatant was poured off carefully, and the pellet was dried at room temperature in a clean laminar flow bench for approximately 1 hour. Subsequently, the pellet was dissolved in prewarmed (40°C) 0.1 x Tris-EDTA buffer for further analysis. RNA was isolated from ground dormant leaf tissue and prepared as described above using a NucleoSpin RNA Plant Kit (Macherey-Nagel, Düren, Germany) according to the protocol supplied with the kit. The extracted DNA and RNA were checked for integrity and quantity using agarose gel electrophoresis and fluorometry on a Qubit v3 device (ThermoFisher, United States), respectively.

#### Sequencing

From genomic DNA shotgun TruSeq paired-end libraries of 300 bp and 600 bp insert lengths and long-jumping-distance (LJD) libraries of 3 kbp, 8 kb, and 20 kb were constructed for paired-end sequencing (2 × 100 bp) on an Illumina HiSeq 2000 Sequencer Iillumina, United States) by a commercial sequencing provider (LGC Genomics GmbH, Germany). In addition, libraries with a target insert size of 20 kb for Single Molecule Real-Time (SMRT) sequencing on a Pacific Biosciences (PacBio) RSII instrument (United States) using the DNA/Polymerase Binding Kit P6 were constructed and sequenced by a commercial sequencing provider (Eurofins Genomics, Germany) using 6 SMRT cells. In addition, both mRNA-enriched and ribosome-depleted TruSeq paired-end libraries were prepared and subsequently sequenced on a HiSeq 2000 instrument by LGC Genomics GmbH (Germany).

#### Assembly and quality control

Illumina reads were checked for adapter sequences and bad-quality read ends using Trimmomatic v0.36 (Trimmomatic, RRID:SCR_011848) [33] with the following parameters: "TruSeq3-PE.fa:2:30:10 LEADING:3 TRAILING:3 SLIDINGWINDOW:4:15 MINLEN:70". Reads with Ns in the sequences were filtered using Sickle (version 1.33) [34]. The final cleaned dataset used included reads with an average quality of more than 30, were longer than 70 bp, and were without Ns. The PacBio reads were corrected by the filtered Illumina reads using Proovread (version 2.14.0) [35], and the corrected reads were further used for the assembly.

All sequencing data as well as the genome assembly can be found under accession number PRJEB24056 at the European Nucleotide Archive (ENA) [36]. The assembly was done using a hybrid assembly approach in which an initial assembly was built using Velvet v.1.2.10 [37] on shotgun reads with insert lengths of 300 bp and 600 bp (35 Gb, corresponding to 75x coverage after adapter trimming and filtering) with a k-mer length of 63 and without scaffolding. This pre-assembly of 360 Mb with a minimum contig length of 300 bp was taken as a base for a DBG2OLC (last update, June 11, 2015) [38] hybrid assembly using corrected PacBio reads >150 nucleotides (7.9 Gb, corresponding to 17x coverage, mean size 9,487 nucleotides, median 9,162 nucleotides, longest sequence 47,053 nucleotides) with a k-mer length of 17, a k-mer matching threshold for each contig of 5, minimum matching k-mers for each two reads of 30, adaptive k-mer threshold for each contig of 0.002, and chimera removal option set to 1. The resulting assembly of 541 Mb was further scaffolded with Illumina LJD libraries using SSpace (basic version) (SSPACE, RRID:SCR_005056) [39]. The genome size was estimated using k-mer counting based on the depth distribution as computed by Jellyfish v 2.0 (Jellyfish, RRID:SCR_005491) [40] using 15-mers and considering all coverage depths using R-scripts.

A CEGMA v 2.5 (CEGMA, RRID:SCR_015055) [41] analysis was performed to test for the completeness and continuity of the beech genome assembly, along with other published tree genomes. In addition, the assembly was evaluated with plant-specific Benchmarking Universal Single-Copy Orthologs (BUSCO, RRID:SCR_015008) [42].

#### Gene prediction

Splice alignments of Illumina RNA sequencing (RNA-seq) data (filtered using the same criteria as above for genomic reads, in total 3.2 Gb) were built using Tophat2 v 2.0.10 (TopHat, RRID:SCR_013035) [43] using the draft genome. This alignment was used in Blast2GO v4.1 (Blast2GO, RRID:SCR_005828) [44] along with a pretrained dataset from *Arabidopsis thaliana*. Genes were predicted on both strands. Genes with a length of more than 90 nucleotides with both a start and a stop codon were considered. For the other parameters, default values were opted. Genes were annotated using Blast2GO. For the sequence similarity-based annotation, a locally downloaded protein-RefSeq database [45] was queried using the Blastp-fast algorithm of the Basic Local Alignment Search Tool (BLAST), version 2.2.30+ (National Center for Biotechnology Information [NCBI] BLAST, RRID:SCR_004870). In a second, less-stringent approach to predict more splice variants, splice-alignment information from RNA-Seq mapping was used along with the single-copy protein sequences predicted in the BUSCO pipeline [42], in the BRAKER2 pipeline (version 2.1.0) [46] using GeneMark-ET v 4.29 [47] and Augustus v3.2.6 (Augustus: Gene Prediction, RRID:SCR_008417) [48]. The splice alignments of RNA-seq reads mapped on the genome were also used as extrinsic evidence in this approach.

#### Repeat prediction

RepeatScout v1.0.5 (RepeatScout, RRID:SCR_014653) [49] was used for *de novo* identification of repeat elements and for generating a repeat element database. This database was used in RepeatMasker v4.0.5 (RepeatMasker, RRID:SCR_012954) [50] to predict repeat elements. Putative repeats were further filtered on the basis of their copy numbers. Those repeats represented with at least 10 copies in the genome were retained.

#### General genomic features

For each annotated gene, the shortest distance to the next gene on the same scaffold was measured. In addition, the distance between all heterozygous sites was assessed, as identified by positions with a two-base ambiguity code in the assembly. For this, genomic reads were mapped using MAQ (version 3) [51], and positions were scored as heterozygous if the frequency of the lesser base was at least 40%. For the aforementioned analyses, the assembly was divided into nonoverlapping windows of 10-kb size. For each of the resulting 50,994 windows, gene density, GC-content, and genetic diversity were determined. Exon density was measured as the proportion of each window annotated as protein-coding and GC content as proportion of G and C bases. Genetic diversity was approximated by the proportion of heterozygous sites in each window. The values were extracted from the assembly and GFF files using custom-made Python scripts, available upon request. Because genome windows in spatial proximity may not represent independent data, each parameter was tested for spatial autocorrelation using Moran's I as test statistics. The relations between the parameters were explored using linear regression models.

#### Screening for contamination

The genic regions of *F. sylvatica* were blasted against two databases, one containing genes from *Arabidopsis thaliana* and the other containing genes from *Fungi* and *Straminipila*, using an e-value cutoff of 10e^−5^ and extracting the top hits. The genic regions having a fungus as the top hit were blasted against the non-redundant database from NCBI [52] to reveal whether these were indeed specific to fungi. Local alignments of the genic regions remaining after this filtering process to the supposed fungal homologs were subsequently manually inspected for the distribution of conserved features.

In addition, the assembled genome was chopped into 300-bp fragments and subjected to analysis with MEGAN (version 5) [53]. The fragmented genome was blasted against the nucleotide database downloaded from NCBI using an e-value cutoff 10e^−8^ and a 70% identity cutoff.

### Data description, validation, and control

#### Genome summary

Raw reads, assembly, and annotations are available from the ENA at accession number PRJEB24056 and at the Beech Genome Resource website [54]. The genome size was estimated to be 541 Mb based on 15-mer counts (Supplementary Fig. S1), while the draft genome assembly was 542 Mb. The assembly comprised 6,491 scaffolds, with 0.12% Ns. The largest scaffold was 1.15 Mb, the N50 length was 145 kb, and the L50 count was 983. Also, 58.36% of the genome is classified as interspersed repeats and around 2% of the genome is comprised of simple repeats. The locations of the interspersed repeats and the simple repeats in the scaffolds are provided as a gff file for download and as a separate track in the genome browser [54]. In total, 62,012 genes and 73 splice variants were predicted using Blast2Go, of which 58,211 genes had received at least one RNA-seq read support ( 50,723 genes were supported by at least five reads). The average number of exons per gene was 4.59, and the distribution of the number of exons per gene was similar to that of other genomes (Supplementary Fig. S2). The BRAKER2-based gene prediction resulted in 100,822 complete genes, including 1,332 splice variants. Of the genes predicted by BRAKER2, 90,936 genes were supported by at least one RNA-seq read ( 73,598 genes were supported by at least five reads). This gene set is given as an additional track in the genome browser and as a supplementary gene annotation file on the genome resource page [54]. A total of 60,879 genes predicted by Blast2GO gene were found to be present in the gene set predicted by BRAKER2 pipeline according to a homology-based sequence similarity analysis using Blastp (version: 2.2.29+) with an e-value cutoff of 10e-10.

**Figure 2:**
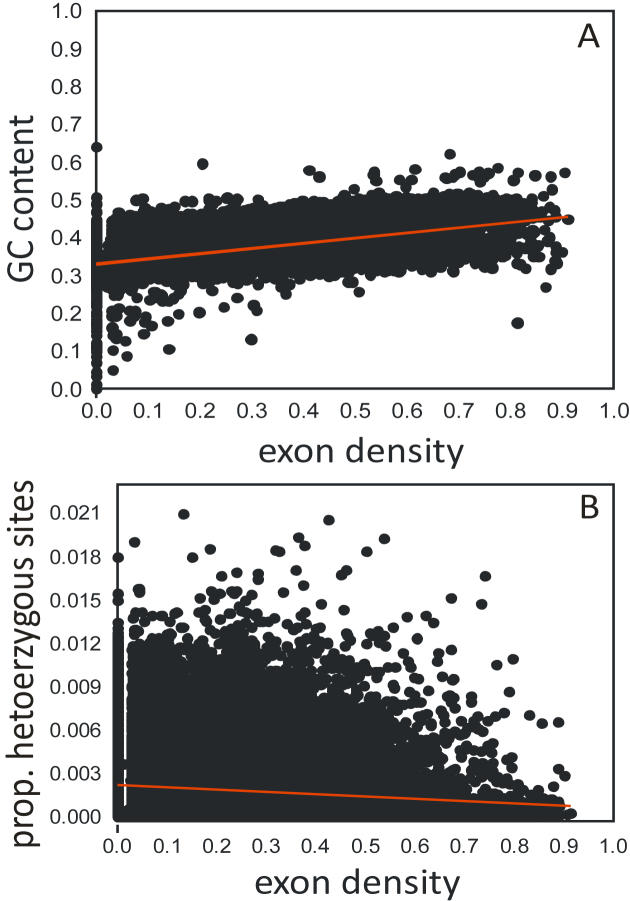
Parameter correlations in the *Fagus sylvatica* genome. **(A)** Gene density vs the GC content in each of the 50,994 nonoverlapping 10-kb windows. (B) Gene density vs. the proportion of heterozygous sites.

The mean (median) minimum observed distance between annotated genes on the same scaffold was 2,696 (1,617) bp, ranging from 1 bp to about 73 kb (Supplementary Fig. S3). The mean (median) distance among neighboring heterozygous sites was 460 (95) bp, with a range of 1 to 136 kb (Supplementary Fig. S4). Gene density in 10-kb windows was between 0 and 0.99 coverage, with a mean (median) of 0.196 (0.170) (Fig. 2A). The respective density values for exons fell to between 0 and 0.87, with a mean of 0.196 and a median of 0.170. The mean (median) GC content of the windows was 0.356 (0.349; Fig. 2B). This is about 5% lower than published values for beech [55] but refers here to only the nonrepetitive regions of the genome. On average, 2 in 1,000 sites were heterozygous (0.0019), with a range of 0 to 0.021.

**Figure 3:**
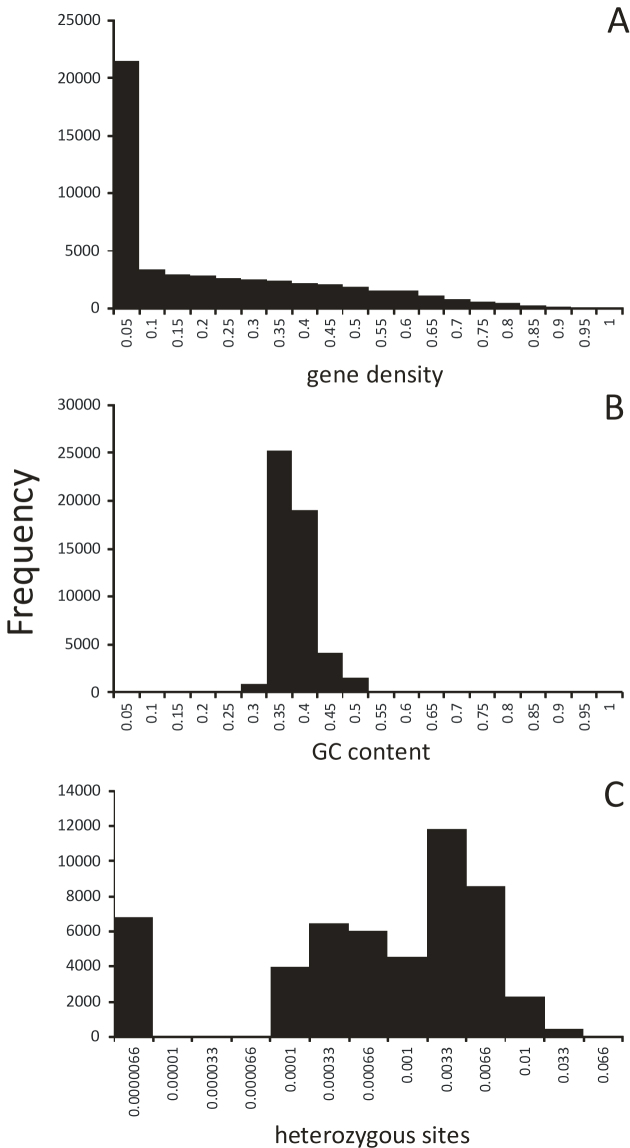
Parameter frequency distributions in 50-994 nonoverlapping 10-kb windows. **(A)** Gene density, measured as proportion of the window annotated as gene. **(B)** Proportion of GC bases. **(C)** Genetic diversity, measured as proportion of heterozygous sites.

Because there was no spatial autocorrelation among adjacent nonoverlapping 10-kb windows or multiples of it (Moran's I <10–4) for either parameter, we could treat the extracted values as independent data points. There was a very strong relationship between exon density and GC content (r² = 0.91, *P* < 0.0001; Fig. 3A), while the correlation between gene density and GC content was marginal (r² = 0.02, *P* < 0.0001). This pattern was observed in many angiosperms and is usually explained as GC-biased gene conversion [56].

Positive, purifying, and background selection on functional genome elements is thought to negatively influence genetic diversity [57]. Therefore, a negative correlation between exon density and genetic diversity could be expected and, albeit very weak, was indeed found (r² = 0.015, *P* < 0.0001; Fig. 3B). This may reflect that adaptation processes in beech affect quantitative, polygenically encoded traits [58], and therefore molecular signatures of selection differ only slightly from neutral expectations [57, 59, 60].

#### Flow cytometric genome size and GC-content estimation

The measured 2C value was 1.191±0.003 pg and the GC content was 37.34%. The between-day variation caused by random instrument drift and/or nonidentical sample preparation did not exceed 0.6%. The GC content and 2C value are in the range of previously reported estimates for *F. sylvatica* (36.7%–39.9%, 1.11–1.30 pg; [55, 61]). Interestingly, when compared to the data from the European distribution of *F. sylvatica* measured from leaves using the same methodology, the studied sample matches with the geographically nearby sample from Gruenewald, Luxembourg [61].

After conversion of the 2C value to number of bases (1 pg = 978 Mb), the 1C genome was calculated to be 582.399 Mb. This value is reasonably close to the draft genome assembly. The difference of approximately 40 Mb can likely be attributed to the collapsing of centromeric and telomeric repeats in the assembly.

#### Genome completeness

The CEGMA analysis for evaluating assembly completeness and continuity showed a high level of completeness, with 242 of 248 (94%) of the Core Eukaryotic Genes (CEGs) at least partially covered, including 213 CEGs (82%) considered complete as per CEGMA criteria [41]. A BUSCO analysis revealed the retrieval of 94% of complete BUSCO genes, of which 19% were duplicated. Only 1.7% of the BUSCO genes were reported as fragmented and 3.6% were reported to be missing from the genome (Table 1). This places the genome among other high-quality draft genomes for tree species. In total, 75.47% of the shotgun reads used in the assembly mapped back to the assembly uniquely and in correct orientation, covering 532 Mb of the assembly.

**Table 1:** Statistics of the completeness of *de novo* genome assembly of *Fagus sylvatica* assessed with CEGMA and BUSCO

Genome	BUSCO complete (in %)	BUSCO duplicated (in %)	BUSCO fragmented (in %)	BUSCO missing (in %)	CEGMA complete (in %)	CEGMA partial (in %)	Reference
*Fagus sylvatica v1.2*	94	19	1.7	3.6	82	94	This study
*Castanea mollisima v 1.1*	91	13	4.2	4.0	77	94	[19]
*Quercus robur v1.0*	92	10	2.7	4.8	81	96	[17]
*Quercus lobata v3.0*	94	11	2.4	3.0	83	98	[62]
*Olea europaea v6.0*	87	19	5.2	7.6	90	96	[30]
*Populus trichocarpa v3.0*	96	17	1.4	2.1	92	97	[63]
*Eucalyptus grandis*	94	5	1.8	4.7	93	100	[64]

#### Checks for contamination

As numerous fungi have been reported to be associated with beech [29], special attention was paid to screen for potential fungal contamination. Gene models of *F. sylvatica* were used as query in a homology-based search using BLAST against two databases, one containing the genes of *Arabidopsis thaliana* and the other containing genes from *Fungi* (both extracted from the NCBI nucleotide database), and revealed 222 genic regions with a fungal organism as the top-hit. When these 222 genes were again used as queries in a homology-based search using BLAST against the NR database from NCBI, eight genes were resolved as still having fungal top hits. These eight genes were manually inspected for the distribution of conservation. As conservation was always below a BLAST alignment score of 200 and conserved features were short, there was no conclusive evidence to support that potential contaminant fungi have impacted the assembly. In a MEGAN analysis of the genome chopped into 300 nucleotide fragments, the fragments were either categorized into flowering plants or left unassigned, suggesting a contamination load below detection threshold.

### Re-use potential

The European beech is arguably one of the most important and iconic hardwood tree species in central Europe, where it forms monospecific stands under optimal growing conditions, outcompeting all other European broad-leaved tree species. Thus, there is a keen interest in the ecological genetics and genomics of the species. With the present genomic resources and the established genome browser, we provide a solid foundation for future investigations, giving the data provided a high re-use potential. In addition, the European beech genome adds to the few tree genomes published so far and is likely to be used in a variety of comparative genomics studies. Furthermore, this data resource build based on the individual “Bhaga,” will be part of a large pan-European consortium studying the genomic adaptation of beech and will serve as the reference genome and a cornerstone for future investigations.

## Availability of supporting data

Raw data and assemblies were deposited in the ENA with the project accession PRJEB24056. In addition, the genome and annotation can be accessed and browsed at www.beechgenome.net. Custom scripts, annotations, and other supporting data are also available from the *GigaScience* GigaDB repository [65].

## Additional files

**Figure S1**. Kmer-based genome size estimation.

**Figure S2**. Percentage of genes plotted against the number of exons in a given gene.

**Figure S3**. Distribution of the minimum distance among annotated genes in base pairs.

**Figure S4**. Distribution of distances among heterozygous sites in base pairs.

## Abbreviations

Blast: Basic Local Alignment Search Tool ; CEG:Core Eukaryotic Genes ; ENA: European Nucleotide Archive; LJD: long-jumping-distance; NCBI: National Center for Biotechnology Information; RNA-seq: RNA sequencing; SMRT: Single Molecule Real-Time

## Competing interests

The authors declare that they have no competing interests.

## Supplementary Material

GIGA-D-18-00026_Original_Submission.pdf

GIGA-D-18-00026_Revision_1.pdf

Response_to_Reviewer_Comments_Original_Submission.pdf

Reviewer_1_Report_(Original_Submission) -- Richard Buggs 2/5/2018 Reviewed

Reviewer_2_Report_(Original_Submission) -- Nathaniel Street 2/19/2018 Reviewed

Reviewer_2_Report_(Revision_1) -- Nathaniel Street 5/13/2018 Reviewed

Reviewer_3_Report_(Original_Submission) -- Charles Hefer 2/26/2018 Reviewed

Supplement Figures
