# Supplementary material for: A reference genome of the European beech (Fagus sylvatica L.)
Source: Gigascience. 2018 May 28;7(6):giy063. doi: 10.1093/gigascience/giy063 (PMC6014182; doi:10.1093/gigascience/giy063)
Supplement: GIGA-D-18-00026_Original_Submission.pdf [file giy063_giga-d-18-00026_original_submission.pdf]

GigaScience

A reference genome of the European Beech (Fagus sylvatica L.)

--Manuscript Draft--

Manuscript Number:	GIGA-D-18-00026
Full Title:	A reference genome of the European Beech (Fagus sylvatica L.)
Article Type:	Data Note
Funding Information:	LOEWE (BiK-F, IPF, TBG) — Prof. Dr. Marco Thines<br>Narodowe Centrum Nauki (PL) (2012/04/A/NZ9/00500) — Prof. Dr. Jaroslaw Burczyk
Abstract:	Background: The European Beech is arguably the most important climax broad-leaved tree species in Central Europe, widely planted for its valuable wood. Here we report the 542 Mb draft genome sequence of an up to 300 year-old individual (Bhaga) from an undisturbed stand in the Kellerwald-Edersee National Park in central Germany.<br>Findings: Using a hybrid assembly approach with Illumina reads with short- and long-insert libraries, coupled with long PacBio reads, we obtained an assembled genome size of 542 Mb, in line with previous flow cytometry measurements. The largest scaffold was of 1.15 Mb, the N50 length was 145 kb, and the L50 count was 983. The assembly contained 0.12 % of Ns. A BUSCO analysis retrieved 94% of complete BUSCO genes, well in the range of other high-quality draft genomes of trees. A total of 62,012 protein-coding genes were predicted, assisted by transcriptome sequencing. In addition, we are reporting an efficient method for extracting high molecular weight DNA from dormant buds, by which contamination by environmental bacteria and fungi was kept at a minimum.<br>Conclusions: The assembled genome is a valuable resource for studying the evolution and past climate change adaptation of beech and will be helpful for identifying genes, e.g. involved in draught tolerance, in order to select and breed individuals to adapt forestry to climate change in Europe. A continuously updated genome browser and download page can be accessed from beechgenome.net, which will include future genome versions of the reference individual Bhaga, as new sequencing approaches develop.
Corresponding Author:	Marco Thines<br><br>Frankfurt am Main, GERMANY
Corresponding Author Secondary Information:	
Corresponding Author's Institution:	
Corresponding Author's Secondary Institution:	
First Author:	Bagdevi Mishra
First Author Secondary Information:	
Order of Authors:	Bagdevi Mishra<br>Deepak Kumar Gupta<br>Markus Pfenninger<br>Thomas Hickler<br>Ewald Langer<br>Bora Nam<br>Juraj Paule<br>Rahul Sharma<br>Bartosz Ulaszewski

Powered by Editorial Manager® and ProduXion Manager® from Aries Systems Corporation

	Joanna Warmbier
	Jaroslaw Burczyk
	Marco Thines
**Order of Authors Secondary Information:**	
**Opposed Reviewers:**	Ivan Scotti, Dr.<br>ivan.scotti@inra.fr<br>Project coordinator of a consortium on beech genomics in which the corresponding author is also a member. He could thus probably not deliver an unbiased assessment of the manuscript.
**Additional Information:**	
**Question**	**Response**
Are you submitting this manuscript to a special series or article collection?	No
**Experimental design and statistics**<br><br>Full details of the experimental design and statistical methods used should be given in the Methods section, as detailed in our Minimum Standards Reporting Checklist. Information essential to interpreting the data presented should be made available in the figure legends.<br><br>Have you included all the information requested in your manuscript?	Yes
**Resources**<br><br>A description of all resources used, including antibodies, cell lines, animals and software tools, with enough information to allow them to be uniquely identified, should be included in the Methods section. Authors are strongly encouraged to cite Research Resource Identifiers (RRIDs) for antibodies, model organisms and tools, where possible.<br><br>Have you included the information requested as detailed in our Minimum Standards Reporting Checklist?	Yes
**Availability of data and materials**<br><br>All datasets and code on which the conclusions of the paper rely must be either included in your submission or deposited in publicly available repositories (where available and ethically appropriate), referencing such data using a unique identifier in the references and in	Yes

Powered by Editorial Manager® and ProduXion Manager® from Aries Systems Corporation

the “Availability of Data and Materials” section of your manuscript.<br><br>Have you have met the above requirement as detailed in our Minimum Standards Reporting Checklist?	

Manuscript

Click here to download Manuscript Mishra_et_al_Data_Note_Beech_Genome_for_submission.doc

A reference genome of the European Beech (*Fagus sylvatica* L.)

Bagdevi Mishra[1,2], Deepak K. Gupta[1,2], Markus Pfenninger[1,3], Thomas Hickler[1,2], Ewald Langer[4], Bora Nam[1,2], Juraj Paule[1], Rahul Sharma[1], Bartosz Ulaszewski[5], Joanna Warmbier[5], Jaroslaw Burczyk[5], Marco Thines[1,2]

[1] Senckenberg Biodiversity and Climate Research Centre (BiK-F), Senckenberg Gesellschaft für Naturforschung, Senckenberganlage 25, D-60325 Frankfurt am Main, Germany

[2] Goethe University, Department for Biological Sciences, Institute of Ecology, Evolution and Diversity, Max-von-Laue-Str. 9, D-60438 Frankfurt am Main, Germany

[3] Institut für Organismische und Molekulare Evolutionsbiologie (iOME), Fachbereich Biologie, Johannes Gutenberg Universität, Gresemundweg 2, 55128 Mainz

[4] University of Kassel, FB 10, Department of Ecology, Heinrich-Plett-Str. 40, D-34132 Kassel, Germany

[5] Kazimierz Wielki University, Department of Genetics, ul. Chodkiewicza 30, 85-064 Bydgoszcz, Poland

Author for correspondence – Marco Thines (m.thines@thines-lab.eu).

Abstract

Background: The European Beech is arguably the most important climax broad-leaved tree species in Central Europe, widely planted for its valuable wood. Here we report the 542 Mb draft genome sequence of an up to 300 year-old individual (Bhaga) from an undisturbed stand in the Kellerwald-Edersee National Park in central Germany.

Findings: Using a hybrid assembly approach with Illumina reads with short- and long-insert libraries, coupled with long PacBio reads, we obtained an assembled genome size of 542 Mb, in line with previous flow cytometry measurements. The largest scaffold was of 1.15 Mb, the N50 length was 145 kb, and the L50 count was 983. The assembly contained 0.12 % of Ns. A BUSCO analysis retrieved 94% of complete BUSCO genes, well in the range of other high-quality draft genomes of trees. A total of 62,012 protein-coding genes were predicted, assisted by transcriptome sequencing. In addition, we are reporting an efficient method for extracting high molecular weight DNA from dormant buds, by which contamination by environmental bacteria and fungi was kept at a minimum.

Conclusions: The assembled genome is a valuable resource for studying the evolution and past climate change adaptation of beech and will be helpful for identifying genes, e.g. involved in draught tolerance, in order to select and breed individuals to adapt forestry to climate change in Europe. A continuously updated genome browser and download page can be accessed from beechgenome.net, which will include future genome versions of the reference individual Bhaga, as new sequencing approaches develop.

Key words – biodiversity, climate change, forest tree, fungi, genomics, hybrid assembly, transcriptomics, tree.

Data description

Context

European Beech (*Fagus sylvatica* L.) is one of the most important and widespread broad-leaved tree species in Europe. Its natural range extends from southern Italy to southern Scandinavia and from the Iberian peninsula to Crimea [1] (San-Miguel-Avanz et al. 2016). Under favourable conditions, in particular in Central Europe, it can outcompete all other tree species and form mono-specific stands, in which, due to shading, other broad-leaved species can hardly establish [2] (Ellenberg and Leuschner 2010). Because of their cultural and environmental importance, as well as their global uniqueness, ancient and primeval beech forests in the Carpathians and five areas in Germany have been listed as UNESCO World Heritage sites [3]. Langer et al. [4] analysed the species composition of these forests and concluded a need for conservation of near natural or primeval beech forest stages for their richness in fungal species.

In total, there have been 1766 fungal species reported associated with beech, ranging from general commensals to specialised pathogens and symbionts, such as the very common obligate mycorrhizal symbiont *Lactarius blennius* (Beech Milkcap), with a distribution corresponding to the natural distribution of beech [5,6]. On average 25 fungal species are associated with dead wood of *F. sylvativa* [7]. Among them are threatened species and species with nature value like *Hericium coralloides* or *Phleogena faginea* [8,9]. Nitrogen uptake by beech roots is highly dependent on the mycorrhizal community [10]. Thus, the European Beech is in intimate contact with a variety of fungi.

Even though its natural area of dominance [11] has been reduced by land use and planting other commercially important species, such as Norway Spruce (*Picea abies*; [12]), it remains an important hardwood species at the European scale. As European beech, however, does not cope very well with dry and hot conditions or fire, and neither with flooding, its suitability under a potentially more extreme climate in the future is debated [13]. Thus, genetic and genomic data are crucial for understanding its adaptive capacity, in particular under climate change [14], with its associated change in biotic stress, including fungal pathogens [15,16].

Several tree genomes have been released over the past decade, among them oaks [17,18] and Chinese Chestnut [19] of the beech family (*Fagaceae*). However, despite its economic and ecological importance, genetic and genomic resources in the genus *Fagus* (beeches) are limited to some studies of the genetic diversity and candidate genes using SNP data [20-23], few genome-wide associations studies [24,25], methylation patterns [26] and some transcriptome data [27,28]. Thus, it was the aim of this study to provide a draft assembly of the European Beech and to make it available to the research community for in-depth analyses and follow-up studies taking advantage of the genomic resource. The risk of contamination with a variety of microorganisms, including bacteria and the numerous fungi found in association with trees in general and beech in particular [29], is high when conducting sampling of specimens from nature, as evidenced by the high amount of contaminant DNA in the effort of sequencing the olive tree genome from an 1000 year-old individual [30]. Thus, we are also describing a method of DNA extraction from dormant buds, which in our case led to the absence of contaminant organisms in the assembly.

Methods

Selection of the sequenced individual

For the genome sequencing, an individual standing on a rocky outcrop on the rim of a scarp to the Edersee (German Kellerwald-Edersee National Park) was selected (Fig. 1). The individual, named Bhaga (the reconstructed common root of the common name of the tree in several European languages), is estimated to be up to 300 years old, based on its poor stand, low branching, as well as bark and stem characteristics. A direct measurement was not possible because the trunk is not fully preserved due to the high age of the individual. An old individual was selected to avoid the influence of modern forestry on the genetic makeup of the individual.

DNA and RNA extraction

A modified protocol based on the standard CTAB method described by [31] was applied. The CTAB extraction buffer consisted of 100 mM Tris-HCl, 20 mM EDTA, 1.4 M NaCl, 2 % CTAB, 0.2 % *ß*-

mercaptoethanol and 2.5 % PVP. For DNA extractions about 100 buds with a few millimetres of the subtending branchlets were cut from twigs of a larger branch, and surface sterilised by gentle shaking for two minutes in 4 % sodium hypochlorite solution containing 0.1 % of Tween. Subsequently, the buds were rinsed with sterile water until no foam formation was evident. Afterwards, the water was poured off and the buds were descaled after cutting off the subtending branchlet with sterile scalpels. The dormant leaf tissue in the buds was ground in liquid nitrogen using a mortar and pestle. A total of 1,200 mg of powdered tissue was distributed to 24 2 ml reaction tubes. Each sample was thoroughly mixed with three 3 mm metal beads in 600 µl of extraction buffer and incubated at 60 °C for 30 minutes. After this, 600 µl of phenol : chloroform : isoamyl alcohol (25:24:1) (PCI) was added and the tubes were gently mixed by inversion. Subsequently, the tubes were centrifuged at 19,000 *g* for 2 minutes. 500 µl of the supernatant were transferred to a new tube and 600 µl of PCI was added. The tubes were centrifuged again for 2 minutes and each 500 µl of the supernatant transferred to a new tube. Subsequently, 15 µl RNase A solution (100 mg/mL) were added to each tube and the tubes were incubated at 37 °C for 30 minutes. After the incubation, 600 µl of chloroform was added and the tubes were gently shaken. Subsequently, the tubes were centrifuged at 19,000 x *g* for 2 minutes. The supernatant of all tubes was transferred to a 45 ml reaction tube. 3 M sodium acetate solution at pH 5.3 (supernatant : 3 M sodium acetate solution = 1 : 0.09) and 100 % ethanol (supernatant : ethanol = 1 : 2) were added to the supernatant and the tube was gently mixed by inversion. Afterwards, it was incubated at -20 °C for 30 minutes and centrifuged at 4,800 g for 3 min at 4 °C. The supernatant was carefully poured off and the pellet was washed with 70% ethanol twice. After a final centrifugation at 4,800 g for 2 min at 4 °C, the supernatant was poured off carefully and the pellet was dried at room temperature in a clean laminar flow bench for approximately 1 h. Subsequently the pellet was dissolved in pre-warmed (40 °C) 0.1 x TE buffer for further analysis. RNA was isolated from ground dormant leaf tissue, prepared as described above, using a NucleoSpin RNA Plant Kit (Macherey-Nagel, Düren, Germany) according to the protocol supplied with the kit. The extracted DNA and RNA was checked for integrity and quantity, using agarose gel electrophoresis and fluorometry on a Qubit v3 device (ThermoFisher, USA), respectively.

Sequencing

From genomic DNA shotgun TruSeq™ paired end libraries of 300 bp and 600 bp insert lengths and long-jumping-distance (LJD) libraries of 3 kbp, 8 kb, and 20 kb were constructed for paired-end sequencing (2x 100 bp) on an Illumina HiSeq 2000 Sequencer (illumina, USA) by a commercial sequencing provider (LGC Genomics GmbH, Germany). In addition, libraries with a target insert size of 20 kb for SMRT-sequencing on a PacBio RSII instrument (Pacific Biosciences, USA), using the DNA / Polymerase Binding Kit P6, were constructed and sequenced by a commercial sequencing provider (Eurofins Genomics, Germany) using 6 SMRT cells. In addition, both mRNA-enriched and ribosome-depleted RNASeq TruSeq™ paired-end libraries and subsequent sequencing were carried out on a HiSeq 2000 instrument by LGC Genomics GmbH, Germany.

Assembly and quality control

Illumina reads were checked for adapter sequences and bad quality read ends using Trimmomatic [32] and reads with Ns in the sequences filtered using Sickle [33]. The final cleaned dataset used included reads with an average quality more than 30, longer than 70 bp and were without Ns. The PacBio reads were corrected by the filtered Illumina reads using Proovread [34] and the corrected reads were further used for the assembly.

All sequencing data as well as the genome assembly can be found under the Accession number PRJEB24056 at ENA [35]. The assembly was done using a hybrid assembly approach in which an initial assembly was built using Velvet v.1.2.10 [36] on shotgun reads with insert lengths of 300 bp and 600 bp (35 Gb, corresponding to 75x coverage after adapter trimming and filtering) with a k-mer length of 63 and without scaffolding. This pre-assembly of 360 Mb with a minimum contig length of 300 bp was taken as a base for a DBG2OLC [37] hybrid assembly using corrected PacBio reads > 150 nucleotides (7.9 Gb, corresponding to 17x coverage, mean size 9487 nucleotides, median 9162 nucleotides, longest sequence 47053 nucleotides) with a k-mer length of 17, a k-mer matching threshold for each contig of 5, minimum matching k-mers for each two reads of 30, adaptive k-mer

threshold for each contig of 0.002 and chimera removal option set to 1. The resulting assembly of 541 Mb was further scaffolded with Illumina LJD libraries using SSpace [38]. The genome size was estimated using k-mer counting based on the depth distribution as computed by Jellyfish [39] using 15-mers, but considering all coverage depths using R-scripts.

A CEGMA v 2.5 [40] analysis was performed to test for the completeness and continuity of the beech genome assembly, along with other published tree genomes. In addition, the assembly was evaluated with plant-specific BUSCO [41].

Gene Prediction

Splice-alignments of Illumina RNA-seq data (filtered using the same criteria as above for genomic reads, in total 3.2 Gb) using the draft genome were built using Tophat2 [42]. This alignment was used in Blast2GO [43] along with pre-trained dataset from *Arabidopsis thaliana*. Genes were predicted on both strands. Genes with a length of more than 90 nucleotides with both a start and a stop codon were considered. Otherwise default values were opted. Genes were annotated using Blast2GO. For the sequence similarity based annotation, a locally downloaded protein-RefSeq database [44] was queried using the Blastp-fast algorithm of BLAST, version: 2.2.30+. In a second less stringent approach, to predict more splice variants, splice-alignment information from RNA-Seq mapping were used along with the single copy protein sequences predicted in the BUSCO pipeline [41], in the BRAKER2 pipeline [45] using GeneMark-ET [46] Augustus [47]. The splice-alignments of RNA-seq reads on the genome were also used as extrinsic evidence in this approach.

General Genomic Features

For each annotated gene, the shortest distance to the next gene on the same scaffold was measured. In addition, the distance between all heterozygous sites was assessed, as identified by positions with a two-base ambiguity code in the assembly; for this, genomic reads were mapped using MAQ [48] and positions were scored as heterozygous, if the frequency of the lesser base was at least 40 %. For the aforementioned analyses, the assembly was divided into non-overlapping windows of 10 kb size.

For each of the resulting 50,994 windows, gene density, GC-content and genetic diversity was determined. Exon density was measured as the proportion of each window annotated as protein-coding, GC-content as proportion of G and C bases. Genetic diversity was approximated by the proportion of heterozygous sites in each window. The values were extracted from the assembly and GFF-files using custom made Python scripts, available upon request. Because genome windows in spatial proximity may not represent independent data, each parameter was tested for spatial autocorrelation, using Moran's I as test statistics. The relations between the parameters were explored using linear regression models.

Screening for contamination

The genic regions of *Fagus sylvatica* were blasted against two databases, one containing genes from *Arabidopsis thaliana* and the other containing genes from *Fungi* and *Straminipila*, using an e-value cut-off of $10e^{-5}$ and extracting the top hits. The genic regions having a fungus as top hit were blasted against the NR database from NCBI [49], to reveal whether these were indeed specific to fungi. Local alignments of the genic regions remaining after this filtering process to the supposed fungal homologs were subsequently manually inspected for the distribution of conserved features.

In addition, the assembled genome was chopped into 300 bp fragments and subjected to analysis with MEGAN [50]. The fragmented genome was blasted against the NT database downloaded from NCBI using an e-value cut-off $10e^{-8}$ and a 70 % identity cut-off.

Data description, validation and control

Genome summary

Raw reads, assembly and annotations are available from the European Nucleotide archive at the accession number PRJEB24056 and at the Beech Genome Resource website [51]. The genome size was estimated to be 541 Mb based on 15-mer counts (Fig. S1), while the draft genome assembly was of 542 Mb. The assembly was distributed over 6491 scaffolds, with 0.12% of Ns. The largest scaffold was of 1.15 Mb, the N50 length was 145 kb, and the L50 count was 983. In total, 62012 genes and 73

splice variants were predicted using Blast2Go. The average amount of exons per gene was 4.59, and the distribution of the amount of exons per gene was similar to other genomes (Fig. S2). The Braker2-based gene prediction resulted in 100822 complete genes, including 1332 splice variants, which are given as an additional track in the genome browser and as a supplementary gene annotation file on the genome resource page [51].

The mean (median) minimum observed distance between annotated genes on the same scaffold was 2696 (1617) bp, ranging from 1 bp to about 73 kb (Fig. S3). The mean (median) distance among neighbouring heterozygous sites was 460 (95) bp, with a range of 1 to 136 kb (Fig. S4). Gene density in 10 kb windows ranged between 0 and 0.99 coverage with a mean (median) of 0.196 (0.170) (Fig. 2A). The respective density values for exons fell between 0 and 0.87 with an average of 0.196, 0.170 (mean and median, respectively). The mean (median) GC content of the windows was 0.356 (0.349, Fig. 2B). This is about 5% lower than published values [52], but refers here only to the high complexity regions of the genome. On average, two in thousand sites were heterozygous (0.0019), with a range from zero to 0.021.

Because there was no spatial autocorrelation among adjacent non-overlapping 10 kb windows or multiples of it (Moran's I < 10-4) for either parameter, we could treat the extracted values as independent data points. There was a very strong relationship between exon density and GC content ($r^2 = 0.91$, $p < 0.0001$, Fig. 3A), while the correlation between gene density and GC content was marginal ($r^2 = 0.02$, $p < 0.0001$). This pattern was observed in many angiosperms and is usually explained as GC biased gene-conversion [53].

Positive, purifying and background selection on functional genomics elements should negatively influence genetic diversity [54]. Therefore, a negative correlation between exon density and genetic diversity was expected and, albeit very weak, indeed found ($r^2 = 0.015$, $p < 0.0001$, Fig. 3B). This may reflect the fact that most adaptation processes in beech affect quantitative, polygenically encoded traits [55], and therefore molecular signatures of selection could differ only slightly from neutral expectations [54,56,57].

Genome completeness

The CEGMA analysis for evaluating assembly completeness and continuity showed a high level of completeness, with a total of 242 out of 248 (94%) of the CEGs at least partially covered, including 213 CEGs (82%) considered complete as per CEGMA criteria [40]. A BUSCO analysis revealed the retrieval of 94% of complete BUSCO genes, out of which 19% were duplicated. Only 1.7% of the BUSCO genes were reported as fragmented and 3.6% were reported to be missing from the genome. This places the genome among other high-quality draft genomes for tree species (Table 1).

Checks for contamination

As numerous fungi have been reported to be associated with beech [29], special attention was paid to screen for potential fungal contamination. Blasting of the gene models of *Fagus sylvatica* against two databases, one containing genes from *Arabidopsis thaliana* and the other containing genes from Fungi, revealed 222 genic regions with a fungal organism as top-hit. When these 222 genes were blasted against the NR database from NCBI, eight out them were resolved as still having fungal top hits. These eight genes were manually inspected for the distribution of conservation. As conservation was always below a blast alignment score of 200 and conserved features were short, there was no conclusive evidence to support that potential contaminant fungi have impacted the assembly. In the MEGAN analysis of the genome chopped into 300 nucleotide fragments, the fragments were either categorised into flowering plants or left unassigned, suggesting a contamination load below disturbance threshold.

Re-use potential

The European Beech is arguably one of the most important and iconic hardwood tree species in Central Europe, where it forms monospecific stands under optimal growing conditions, outcompeting all other European broad-leaved tree species. Thus, there is a keen interest in the ecological genetics and genomics of the species. With the present genomic resources and the established genome

browser, we provide a solid foundation for future investigations, giving the data provide a high re-use potential. In addition, the European Beech genome adds to the few tree genomes published so far and is likely to be used in a variety of comparative genomics studies. Furthermore, this data resource build based on the individual ‘Bhaga’, being a part of a large pan-European consortium studying the genomic adaptation of beech will thus serve as the reference genome and a cornerstone for future investigations.

Availability of supporting data

Raw data and assemblies were deposited in the European Nucleotide Archive with the project accession PRJEB24056. In addition, the genome and annotation can be accessed and browsed at www.beechgenome.net.

Declarations

Consent for publication

Not applicable.

Competing interests

The authors declare that they have no competing interests.

Funding

This project was partially supported by LOEWE, in the framework of BiK-F (MP, MT, TH), IPF (MT), and TBG (MP, MT). JB, BU and JW were supported by grant No 2012/04/A/NZ9/00500 from National Science Center, Poland.

Authors' contributions

MT conceived the project. MT and BN collected samples, JP conducted experiments, BN extracted genomic DNA and RNA. BM, DKG and RS assembled the genome, provided annotations and set up the genome browser. BM, BU, DKg, JW, MP, MT analysed the genome, BM, EL, JB, MP, MT, TH wrote the manuscript, with contributions from the other authors. All authors read and approved the final manuscript.

Acknowledgements

The Kellerwald-Edersee National Park is gratefully acknowledged for allowing the sequencing of the individual Bhaga.

References

[1] San-Miguel-Ayanz J, de Rigo D, Caudullo G, Houston Durrant T, Mauri A. European Atlas of Forest Tree Species. Publication Office of the European Union, Luxembourg. 2016. ISBN: 978-92-79-36740-3.

[2] Ellenberg H, Leuschner C. Vegetation Mitteleuropas mit den Alpen, 6th Edition. Eugen Ulmer KG, Stuttgart; 2010.

[3] UNESCO: UNESCO World Heritage sites. http://whc.unesco.org/en/list/ (2017). Accessed 14 Dec 2017.

[4] Langer E, Langer G, Popa F, Rexer K-H, Striegel M, Ordynets A, et al. Naturalness of selected European beech forests reflected by fungal inventories: a first checklist of fungi of the UNESCO World Natural Heritage Kellerwald-Edersee National Park in Germany. Mycol Prog. 2015;14:102.

[5] Pena R. Functional diversity of beech (Fagus sylvatica L.) ectomycorrhizas with respect to nitrogen nutrition in response to plant carbon supply. Cuviller Verlag, Göttingen; 2011.

[6] Farr DF, Rossman AY. Fungal Databases, U.S. National Fungus Collections, ARS, USDA. https://nt.ars-grin.gov/fungaldatabases/ (2017). Accessed 18 Dec 2017.

[7] Heilmann-Clausen J, Aude E, Christensen M. Cryptogam communities on decaying deciduous wood – does tree species diversity matter? Biodiv Cons. 2005;14:2061–2078.

[8] Ódor P, Heilmann-Claussen J, Christensen M, Aude E, Van Dort KW, Piltaver A, Siller I, Veerkamp MT, et al. Diversity of dead wood inhabiting fungal and bryophyte assemblages in semi-natural beech forests in Europe. Biol Cons. 2006;131:58–71.

[9] Christensen M, Heilmann-Claussen J, Walleyn R, Adamčik S. Wood-inhabiting fungi as indicators of nature value in European beech forests. Monitoring and Indicators of Forest Biodiversity in Europe - From Ideas to Operationality. EFI Proceedings No. 51; 2004.

[10] Leberecht M, Dannemann, M, Gschewndtner S, Bilela S, Meier R, Simon J, et al. Ectomycorrhizal Communities on the roots of two beech (Fagus sylvatica) populations from contrasting climates differ in nitrogen acquisition in a common environment. Appl Env Microbiol. 2015;81:5957–5967.

[11] Bohn U, Neuhäusle R, Gollub G, Hettwer C, Neuhäuslová Z, Raus T, et al. Map of the natural vegetation of Europe. Landwirtschaftsverlag Münster; 2003.

[12] Brus D, Hengeveld G, Walvoort D, Goedhart P, Heidema A, Nabuurs G, Gunia K. Statistical mapping of tree species over Europe. Europ J Forest Res. 2012;131:145–157

[13] Gessler A, Keitel C, Kreuzwieser J, Matyssek R, Seiler W, Rennenberg H. Potential risks for European beech (*Fagus sylvatica* L.) in a changing climate. Trees. 2007;21:1–11.

[14] Kramer K, Degen B, Buschbom J, Hickler T, Thuiller W, Sykes MT, de Winter W. Modelling exploration of the future of European beech (*Fagus sylvatica* L.) under climate change - Range, abundance, genetic diversity and adaptive response, Forest Ecol Manage. 2010;259:2213–2222.

[15] La Porta N, Capretti P, Thomsen IM, Kasanen R, Hietala AM, von Weissenberg K. Forest pathogens with higher damage potential due to climate change in Europe. Can J Pl Pathol. 2008;30:177–195.

[16] Lindner M, Maroschek M, Netherer S, Kremer A, Barbati A, Garcia-Gonzalo J, et al. Climate change impacts, adaptive capacity, and vulnerability of European forest ecosystems. Forest Ecol Manag. 2010;259:698–709.

[17] Plomion C, Aury JM, Amselem J, Alaeitabar T, Barbe V, Belser C, et al. Decoding the oak genome: public release of sequence data, assembly, annotation and publication strategies. Mol Ecol Res. 2016;16:254–265.

[18] Sork VL, Fitz-Gibbon ST, Puiu D, Crepeau M, Gugger PF, Sherman R, et al. First Draft Assembly and Annotation of the Genome of a California Endemic Oak *Quercus lobata* Née (Fagaceae). G3. 2016;6:3485–3495.

[19] Hardwood Genomics Project: Castanea mollisima. https://www.hardwoodgenomics.org/chinese-chestnut-genome. Accessed 20 Nov 2017.

[20] Lalagüe H, Csilléry K, Oddou-Muratorio S, Safrana J, de Quattro C, Fady B, et al. Nucleotide diversity and linkage disequilibrium at 58 stress response and phenology candidate genes in a European beech (*Fagus sylvatica* L.) population from southeastern France. Tree Gen Genomes. 2014;10:15–26.

[21] Csilléry K, Lalagüe H, Vendramin GG, González-Martínez SC, Fady B, Oddou-Muratorio S. Detecting short spatial scale local adaptation and epistatic selection in climate-related candidate genes in European beech (*Fagus sylvatica*) populations. Mol Ecol. 2014;23:4696–4708.

[22] Müller M, Seifert S, Finkeldey R. A candidate gene-based association study reveals SNPs significantly associated with bud burst in European beech (Fagus sylvatica L.). Tree Gen Genomes. 2015;11:116.

[23] Krajmerová D, Hrivnák M, Ditmarová Ľ, Jamnická G, Kmeť J, Kurjak D, Gömöry D. Nucleotide polymorphisms associated with climate, phenology and physiological traits in European beech (*Fagus sylvatica* L.). New Forests. 2017;48:463–477.

[24] Pluess AR, Frank A, Heiri C, Lalagüe H, Vendramin GG, Oddou-Muratorio S. Genome–environment association study suggests local adaptation to climate at the regional scale in *Fagus sylvatica*. New Phytologist. 2016;210:589–601.

[25] Ćalić I, Koch J, Carey D, Addo-Quaye C, Carlson JE, Neale DB. Genome-wide association study identifies a major gene for beech bark disease resistance in American beech (*Fagus grandifolia* Ehrh.). BMC Genomics. 2017;18:547.

[26] Hrivnák M, Krajmerová D, Frýdl J, Gömöry D. Variation of cytosine methylation patterns in European beech (*Fagus sylvatica* L.). Tree Gen Genomes. 2016;13:117.

[27] Lesur I, Bechade A, Lalanne C, Klopp C, Noirot C, Leplé JC, ... & Le Provost G. A unigene set for European beech (Fagus sylvatica L.) and its use to decipher the molecular mechanisms involved in dormancy regulation. Mol Ecol Res. 2015;15:1192–1204.

[28] Müller M, Seifert S, Lübbe T, Leuschner C, Finkeldey R. De novo transcriptome assembly and analysis of differential gene expression in response to drought in European Beech. PloS one. 2017;12:e0184167.

[29] Unterseher M, Peršoh D, Schnittler M. Leaf-inhabiting endophytic fungi of European Beech (*Fagus sylvatica* L.) co-occur in leaf litter but are rare on decaying wood of the same host. Fungal Div. 2013;60:43–54.

[30] Cruz F, Julca I, Gómez-Garrido J, Loska D, Marcet-Houben M, Cano E, et al. Genome sequence of the olive tree, *Olea europaea*. GigaScience. 2016;5:29.

[31] Doyle JJ, Doyle JL. A rapid DNA isolation procedure for small quantities of fresh leaf tissue. Phytochem Bull. 1987;19:11–15.

[32] Bolger AM, Lohse M, Usadel B. Trimmomatic: a flexible trimmer for Illumina sequence data. Bioinformatics. 2014;30:2114–2120.

[33] Joshi NA, Fass JN. Sickle: A sliding-window, adaptive, quality-based trimming tool for FastQ files (Version 1.33). https://github.com/najoshi/sickle (2015). Accessed 10 April 2016.

[34] Hackl T, Hedrich R, Schultz J, Förster F. proovread: large-scale high-accuracy PacBio correction through iterative short read consensus. Bioinformatics. 2014;30:3004–3011.

[35] EBI: European Nucleotide Archive. https://www.ebi.ac.uk/ena (2017). Accessed 14 Dec 2017.

[36] Zerbino DR and Birney E. Velvet: algorithms for de novo short read assembly using de Bruijn graphs. Genome Res. 2008;18: 821–829.

[37] Ye C, Hill CM, Wu S, Ruan J, Ma ZS. DBG2OLC: efficient assembly of large genomes using long erroneous reads of the third generation sequencing technologies. Sci Rep. 2016;6:31900.

[38] Boetzer M, Henkel CV, Jansen HJ, Butler D, Pirovano W. Scaffolding pre-assembled contigs using SSPACE. Bioinformatics. 2010;27:578–579.

[39] Marcais G, Kingsford C. A fast, lock-free approach for efficient parallel counting of occurrences of k-mers. Bioinformatics 2011;27:764–770

[40] Parra G, Bradnam K, Korf I. CEGMA: a pipeline to accurately annotate core genes in eukaryotic genomes. Bioinformatics. 2007;23:1061–1067.

[41] Simão FA, Waterhouse RM, Ioannidis P, Kriventseva EV, Zdobnov EM. BUSCO: assessing genome assembly and annotation completeness with single-copy orthologs. Bioinformatics. 2015;31:3210–3212.

[42] Kim D, Pertea G, Trapnell C, Pimentel H, Kelley R, Salzberg SL. TopHat2: accurate alignment of transcriptomes in the presence of insertions, deletions and gene fusions. Genome Biol. 2013;14:R36.

[43] Conesa A, Götz S, García-Gómez JM, Terol J, Talón M, Robles M. Blast2GO: a universal tool for annotation, visualization and analysis in functional genomics research. Bioinformatics. 2005;21:3674–3676.

[44] NCBI: RefSeq database. ftp://ftp.ncbi.nlm.nih.gov/blast/db/ (2017 December 30th, 2017)

[45] Hoff J. BRAKER2. http://bioinf.uni-greifswald.de/augustus/binaries/BRAKER2.tar.gz (2017). Accessed 10 Dec 2017.

[46] Lomsadze A, Burns PD, Borodovsky M. Integration of mapped RNA-Seq reads into automatic training of eukaryotic gene finding algorithm. Nucleic Acids Res. 2014;42:e119.

[47] Stanke M, Waack S. Gene prediction with a hidden Markov model and a new intron submodel. Bioinformatics 2003;19(Suppl 2):II215–II225.

[48] Li H, Ruan J, Durbin R. Mapping short DNA sequencing reads and calling variants using mapping quality scores. Genome Res. 2008;18:1851–1858.

[49] NCBI: NR database ftp://ftp.ncbi.nlm.nih.gov/blast/db/ (2017). Accessed 30 Jun 2017.

[50] Huson DH, Beier S, Flade I, Górska A, El-Hadidi M, Mitra S, et al. MEGAN Community Edition – Interactive Exploration and Analysis of Large-Scale Microbiome Sequencing Data. PLoS Comp Biol. 2016;12:e1004957.

[51] Mishra B, Gupta DK, Thines M. The Beech Genome Online Resource (BeGOR). http://www.beechgeneome.net (2017). Accessed 10 Dec 2017.

[52] Gallois A, Burrus M, Brown S. Evaluation of the nuclear DNA content and GC percent in four varieties of *Fagus sylvatica* L. Ann Forest Sci. 1999;56:615–618.

[53] Glémin S, Clément Y, David J, Ressayre A. GC content evolution in coding regions of angiosperm genomes: a unifying hypothesis. Trends Gen. 2014;30: 263–270.

[54] Charlesworth B. Why we are not dead one hundred times over. Evolution 2013;67:3354–3361.

[55] Gömöry D, Ditmarová Ľ, Hrivnák M, Jamnická G, Kmeť J, Krajmerová D, Kurjak D. Differentiation in phenological and physiological traits in European beech (*Fagus sylvatica* L.). European J Forest Res. 2015;134:1075–1085.

[56] Messer PW, Ellner SP, Hairston NG. Can population genetics adapt to rapid evolution? Trends Gen 2016;32:408–418.

[57] Charlesworth B. Effective population size and patterns of molecular evolution and variation. Nature Rev Gen. 2009;10: 195–205.

[58] Valley Oak Genome Project. Quercus mollisima assembly v3. https://valleyoak.ucla.edu/genomicresources (2017). Accessed on the 8 Dec 2017.

[59] Tuskan GA, Difazio S, Jansson S, Bohlmann J, Grigoriev I, Hellsten U, et al. The genome of black cottonwood, *Populus trichocarpa* (Torr. & Gray). Science. 2006;313:1596–1604.

Table caption

Table 1. Statistics of the completeness of de novo genome assembly of *Fagus sylvatica* assessed with CEGMA and BUSCO

Figure captions

Figure 1. Photograph of the sequenced individual Bhaga at time of sampling. Note the very low branching on the cliff, with a major part of the individual reaching over the edge.

Figure 2. Parameter correlations in the *Fagus sylvatica* genome. A: gene density versus the GC content in each of the 50994 non-overlapping 10kb windows. B: gene density versus the proportion of heterozygous sites.

Figure 3. Parameter frequency distributions in 50994 non-overlapping 10 kb windows. A: gene density, measured as proportion of the window annotated as gene. B: proportion of GC bases. C: genetic diversity, measured as proportion of heterozygous sites.

Figure S1. Kmer-based genome size estimation.

Figure S2. Percentage of genes plotted against the number of exons in a given gene.

Figure S3. Distribution of the minimum distance among annotated genes in base pairs.

Figure S4. Distribution of distances among heterozygous sites in base pairs.

Table Click here to download Table Table_01.docx

Table 1. Statistics of the completeness of de novo genome assembly of *Fagus sylvatica* assessed with CEGMA and BUSCO

Genome	BUSCO complete (in %)	BUSCO duplicated (in %)	BUSCO fragmented (in %)	BUSCO missing (in %)	CEGMA complete (in %)	CEGMA partial (in %)	Reference
Fagus sylvatica v1.2	94	19	1.7	3.6	82	94	This study
Castanea mollisima v 1.1	91	13	4.2	4.0	77	94	[19]
Quercus robur v1.0	92	10	2.7	4.8	81	96	[17]
Quercus lobata v3.0	94	11	2.4	3.0	83	98	[58]
Olea europaea v6.0	87	19	5.2	7.6	90	96	[30]
Populus trichocarpa v3.0	96	17	1.4	2.1	92	97	[59]

Figure Click here to download Figure Figure_01.JPG

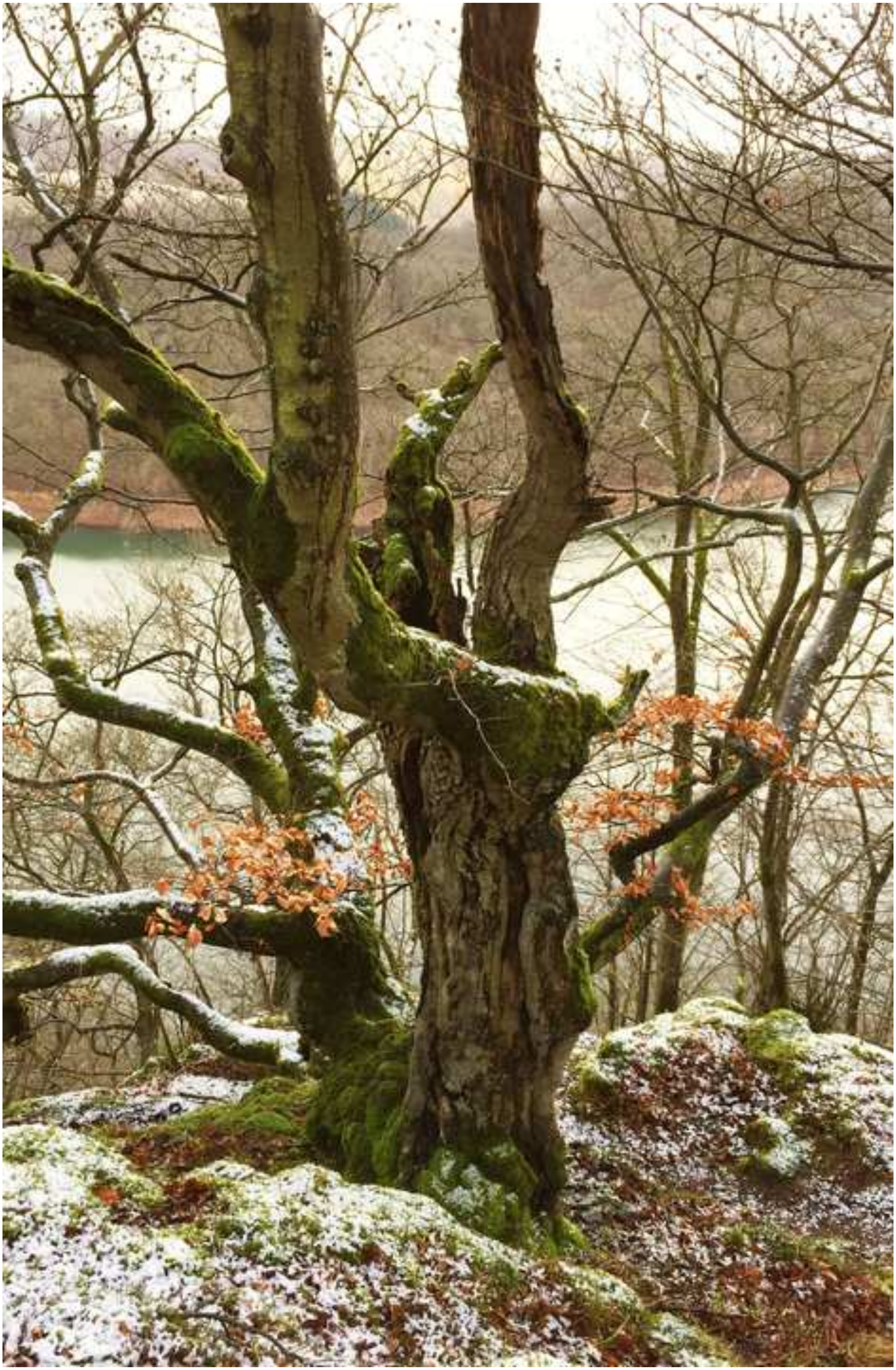

Figure

Click here to download Figure Figure_02.ppt

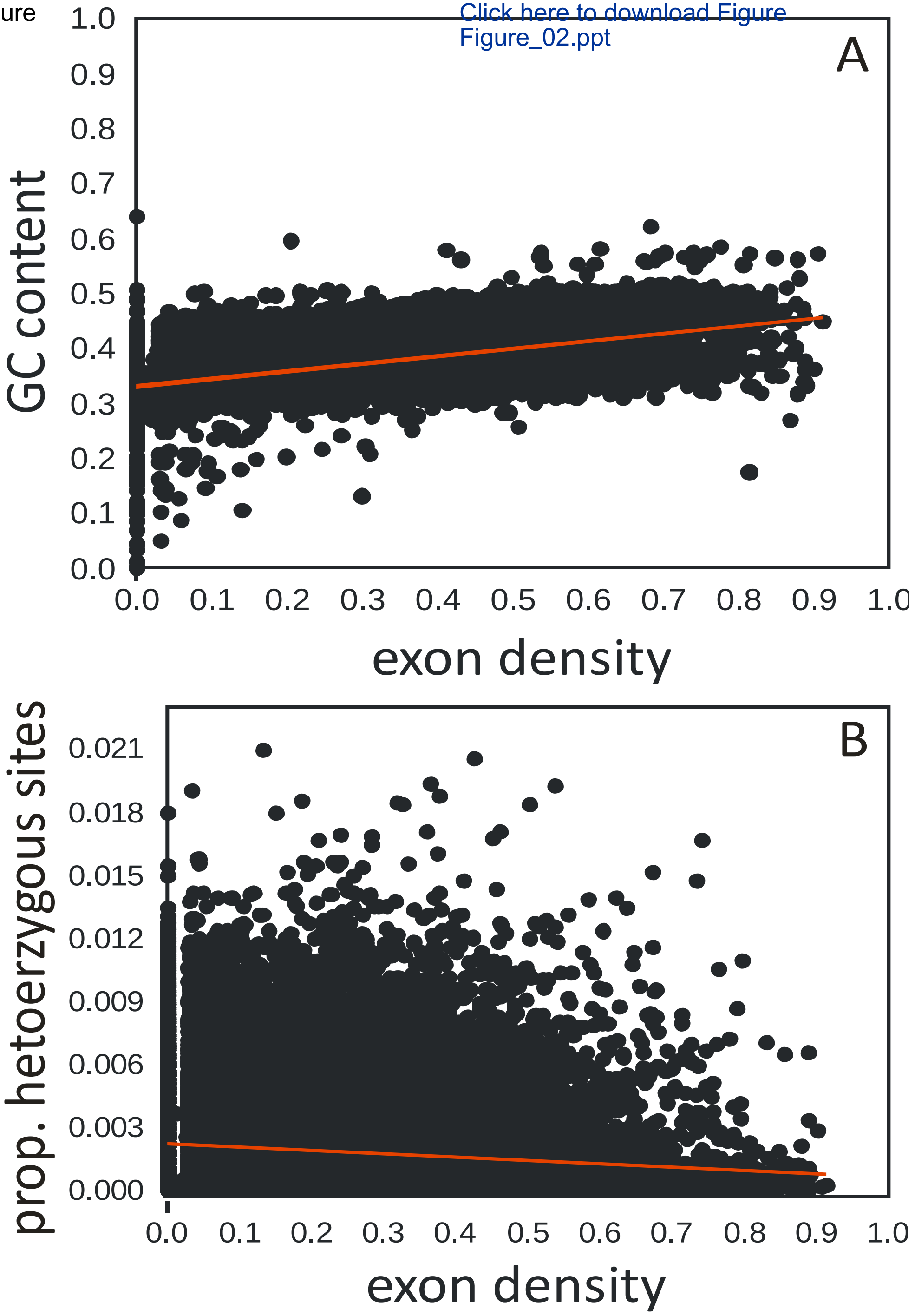

Figure

Click here to download Figure Figure_03.ppt

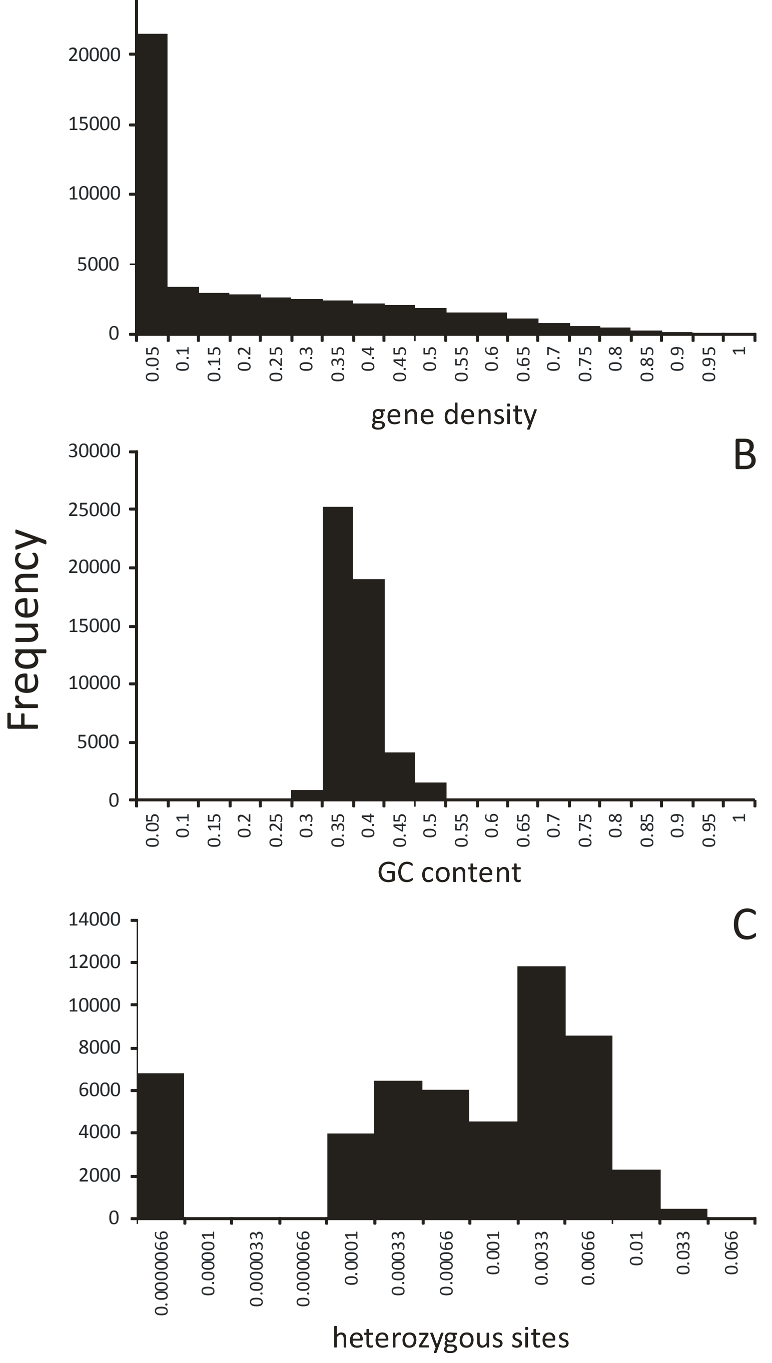

Click here to access/download
Supplementary Material
Figure_S1.pptx

Click here to access/download
Supplementary Material
Figure_S2.pptx

Click here to access/download
Supplementary Material
Figure_S3.ppt

Click here to access/download
Supplementary Material
Figure_S4.ppt
