# Supplementary material for: A reference genome of the European beech (Fagus sylvatica L.)
Source: Gigascience. 2018 May 28;7(6):giy063. doi: 10.1093/gigascience/giy063 (PMC6014182; doi:10.1093/gigascience/giy063)
Supplement: Supplement Figures [file giy063_supplement_figures.zip › Figure_S2.pptx]

## Slide 1
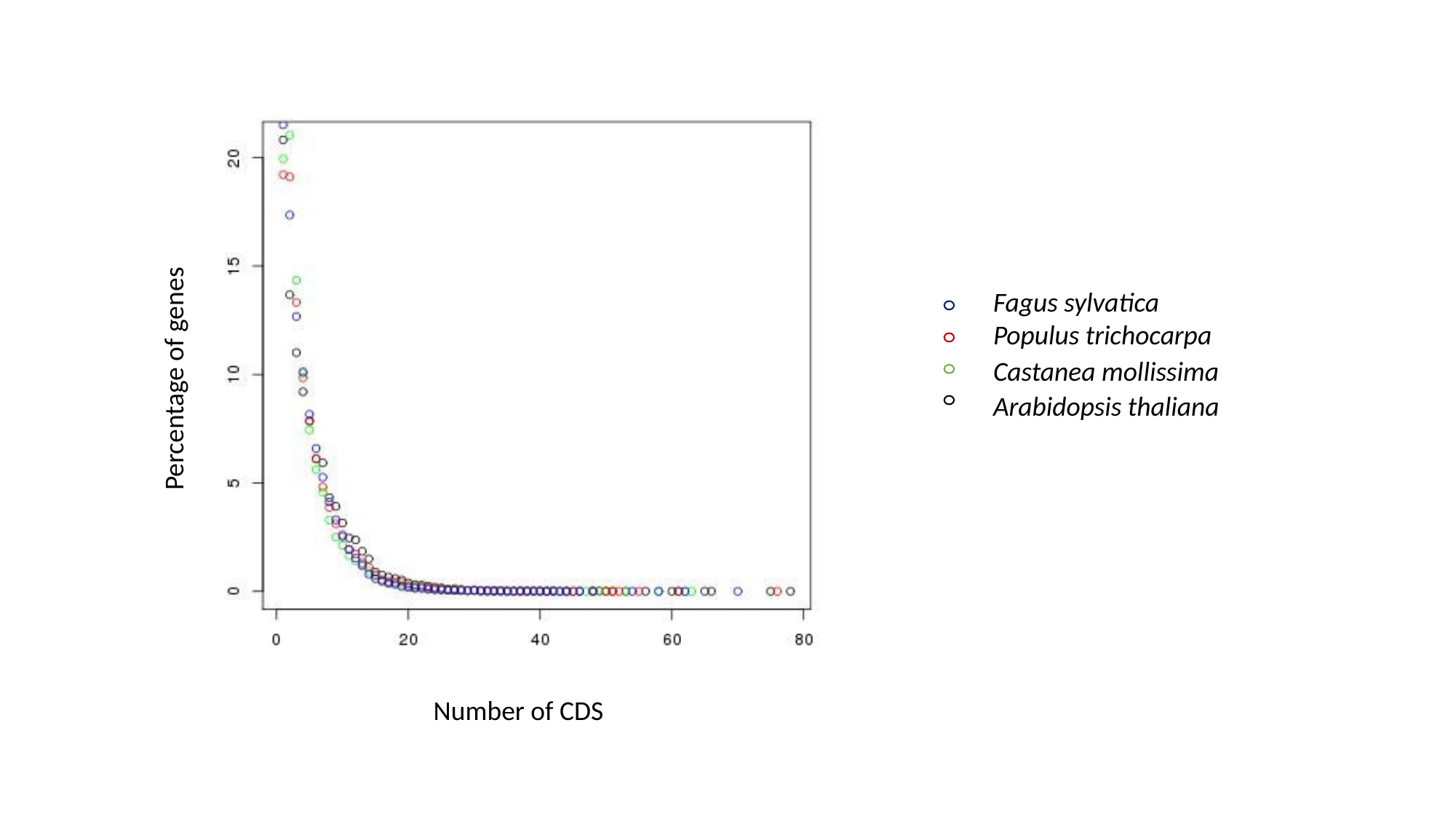

Fagus sylvatica
Populus trichocarpa
Castanea mollissima
Percentage of genes
Arabidopsis thaliana
Number of CDS
