# Supplementary figures and images for: A reference genome of the European beech (Fagus sylvatica L.)
Source: Gigascience. 2018 May 28;7(6):giy063. doi: 10.1093/gigascience/giy063 (PMC6014182; doi:10.1093/gigascience/giy063)

## Slide 1
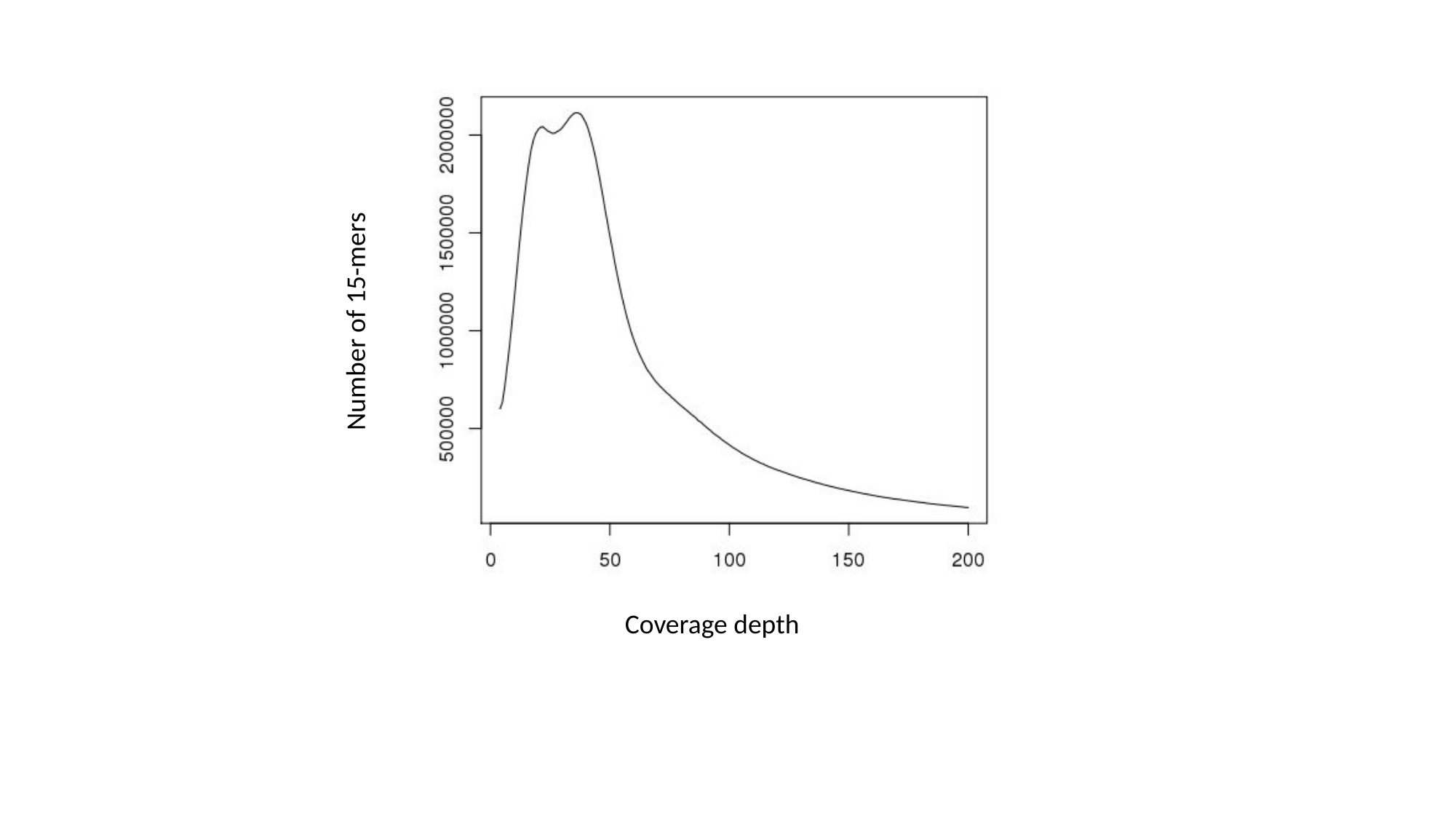

Number of 15-mers
Coverage depth

Supplement: Supplement Figures [file giy063_supplement_figures.zip › Figure_S1.pptx]
