# Supplementary material for: A reference genome of the European beech (Fagus sylvatica L.)
Source: Gigascience. 2018 May 28;7(6):giy063. doi: 10.1093/gigascience/giy063 (PMC6014182; doi:10.1093/gigascience/giy063)
Supplement: Response_to_Reviewer_Comments_Original_Submission.pdf [file giy063_response_to_reviewer_comments_original_submission.pdf]

Author's Response To Reviewer Comments

Close

Reply to the comments of the reviewers

Your manuscript "A reference genome of the European Beech (Fagus sylvatica L.)" (GIGA-D-18-00026) has been assessed by our reviewers. Although it is of interest, we are unable to consider it for publication without a little additional work. The reviewers have raised a number of points which we believe would improve the manuscript and would allow a revised version to be published in GigaScience.

Our Data Note articles do not require analysis but do require sufficient validation and benchmarking, so please make sure the data is compared to all related publicly available genome sequences.
All available Fagaceae genomes and representative tree genomes have been included (with the addition of Eucalyptus, as suggested by reviewer 3).
We have added additional benchmarks as suggested by reviewer 2.

Reviewer #1: This paper reports a good Illumina-Pacbio hybrid assembly for a European Beech tree. This is an important addition to our knowledge of plant genomics.

In line 34, "draught" should be "drought"
Corrected.

Lines 46 and 48 - reference formatting issues
Corrected.

I find lines 212 to 216 hard to follow. Are the previously published values for the whole genome? How were they derived? How are the authors defining "high complexity regions". I suggest these sentences are re-written to make them clearer.
Rephrased.

Reviewer #2: Mishra et al. present the draft assembly of European beech. A very superficial and dry analysis is reported of basic assembly features. There is no repeat annotation, no assembly correctness assessment and a relatively unusual approach to gene annotation that presents potential users with two highly contrasting gene annotations that have not been merged or compared.
We have now added repeat annotation.
Busco already provides a quite decent assembly correctness estimate. In addition, we have now provided information of how many paired reads used for the assembly mapped back to the genome in the correct orientation.
We consider the Blast2Go annotations to represent the high confidence gene set, as mentioned in the manuscript. The Breaker2 pipeline output is just added as a track in the genome browser for experienced users, as it covers additional potential genes, especially those in repeat regions. The vast majority of the genes predicted by Blast2Go are also found in the Breaker2 prediction. This value was now added.

For example, the BREAKER analysis identified almost twice as many genes as the BLAST2GO analysis - what are all those extra genes?
See above.

Very little use is made of the RNA-Seq data, which is extremely limited. There is no presented analysis of how many genes were supported by RNA-Seq evidence and no way of ascertaining what, if anything, this single RNA-Seq sample contributed.
As stated in the manuscript, the RNA-Seq data were intrumental in gene predictions. We have now added the figures regarding how many genes were supported by RNA-Seq data in both gene sets.

Why were no analyses of gene families presented, for example to look for expanded gene families involved in fungal interactions? The presented results lack any biological insight or analysis and the detailed assembly characteristics are of limited to no interest.
We strongly disagree. The purpose of the Data Note format of GigaScience is not to provide detailed analyses already, but to provide the data to the community. And as the study is part of a large consortium effort on beech population genomics, we know that the resource is in fact of great interest. Detailed analyses on various aspects of the genome will follow both by our group as well as other groups that have already expressed interest in the data.

The authors should carefully check the manuscript, particularly the use of commas. There are a number of cases where a closing comma is required, making some sentences hard to read. However, the manuscript is generally clear and concise.
We have thoroughly proofread the manuscript, again.

As there is nothing novel, new or different to the DNA extraction employed for this work I suggest that reference to this be removed from the abstract.
We disagree. Most tree genome assemblies suffer from contamination, as mature leaves were used. Our approach, using the well-shielded dormant buds, has yielded virtually contamination-free DNA. Thus, this approach will very likely be useful for future sequencing efforts.

Although the annotation and analysis of the presented assembly are far from comprehensive, I see no obvious errors or problems with the described methodology.
We are grateful for this positive assessment.

However, some further exploration of assembly quality at each step of the assembly would have been very useful for informing potential users as to the reliability of the assembly. There are a number of tools for performing such analyses using alignments of paired-end and jumping libraries. I would very much have liked to see this as it is far from clear whether the presented hybrid approach to combine the Illumina short read and PacBio read data was optimal and how successful this was.
Some additional quality tests were done, as mentioned above.

The authors do not state any justification for the selected methodology or indicate whether other options were explored. Was the combination of tools used an effectively ad hoc approach or were these informed choices?
We have long-standing experience in genome sequencing and assembly strategies and the one we chose was the most efficient one balancing sequencing costs and assembly quality.

I confirm that the web resource linked to is functional, although it is of limited use and functionality.
Thanks for testing

Abstract:

Is the species important because it is a climax species in natural forests, because of its high value in planted stands or both?
Both.

Mb should be Mb pans similarly for all Base Pair units stated throughout.
As a sequence is always in bases not base pairs, we would like to keep Mb when referring to a sequence.

In it a little odd to use BUSCO as if it is a common-use term in the abstract. It would be better in the abstract to say a set of benchmark eukaryotic conserved genes or similar. The conclusions section of the abstract is widely speculative, especially as there are no actual biological analyses presented in the study to support any of these claims.
Suggestion regarding Busco taken, even though we feel it is a quite widely used benchmark. The conclusions are rather an outlook on the things to come. We have rephrased this a bit, but would like to keep the main message (on which grounds the sequencing of a few dozen additional individuals has been funded).

Keywords: It seems strange to list biodiversity and climate change as keywords for the sequencing of a single individual.
Deleted.

Why are two citations styles used simultaneously?
Corrected throughout.

L65 This often-stated need for genomics data is a stretch. How will this genome sequence provide clear and immediate evidence about whether this species will cope with future climate conditions? Such tenuous justifications for the work are really not needed.
This actually IS the justification, on which basis currently many additional individuals are being sequenced.

L92 The authors claim to present a method for extracting contaminant-free DNA. What they actually did was to sample a dormant tissue that happens to have low microbiome abundance. There is nothing novel or unusual about this as a method. It would be far more appropriate to simply state that a tissue type with low abundance of bacteria and fungi was used for the DNA extraction.
We disagree (see earlier statement). If the reviewer would be running a MEGAN analysis over some published tree genomes he might agree.

L93 Define CTAB and similarly always define abbreviations at first use.
Done.

L95 When were the buds sampled?
In February 2015. This information was added.

L117 It seems rather a strange choice to extract RNA to support gene annotation using only a dormant tissue type.
Genes active in dormant tissue are not special. Interestingly, as no genes are drastically upregulated, a low level of constitutive expression can be found for a very wide set of genes.

L135 Here, and throughout, please state the versions of software used and all relevant parameters, stating default where appropriate.

Information added.

L144 How was this k-mer length selected? It is relatively high. What is the expected heterozygosity of beech as this interacts with k-mer length to affect assembly outcome. I also do not understand using a long k-mer here and then a much shorter k-mer length for the hybrid assembly.
This approach has shown to yield the best overall assemblies.

L157 The gene annotation approach is rather unusual. Why were no ab initio or evidence-based annotation pipelines applied? The annotation as presented does not appear to be particularly comprehensive and would miss genes not expressed or not represented in the undefined Arabidopsis dataset.
This assessment is not correct. Both Blast2Go and BRAKER2 use both ab initio and evidence-based gene predictions. This approach would also neither miss genes not expressed nor not present in Arabidopsis.

L159 Were the intron size settings for TopHat2 adjusted to reflect plant species?
Intron size settings were minimum of 50 and maximum of 500000, values for TopHat which in previous assemblies gave reliable results.

L160 What is a pre-trained dataset?
Blast2GO has datasets pre-trained with data from various species enabling to start from an organism a related to the one for which the genes are being predicted.

L162 What does 'Otherwise default values were opted' mean? What is this referring to?
Corrected - This means “For the other parameters, default values were opted.”

L172 I find the described methodology for locating heterozygous positions hard to follow. Were SNPs called using the reads alignments or did this reply only on called sites from the assembly? It is far more common to align reads and to then use a tool such as GATK to call heterozygous positions.
Yes, the reported numbers are on the basis of aligning reads to the already assembled genome and considering the base variation from this alignment.

L187 It is not clear what a BLAST search against Fungi means here? What is the input, exactly, to construct the BLAST index used for this sequence homology search? I also do not understand the logic and why this search was not directly performed to the NCBI NR database.
The sequence id of all the sequence records that belonged to Fungi were listed in a file and this file was used with the option –gilist in the command line blast. In a general NR blast, it is possible that some genes of fungal origin could are listed as plant, e.g. when derived from environmental sequencing. To avoid this, we did blast search into a subset of only fungal genes and Arabidopsis genes separately. And the sequences that did not hit to Arabidopsis but to fungal genes were considered of potential fungal origin.

L200 k-mer based genome size estimates can be very inaccurate. Are there any flow-cytometry measures available?
Flow cytometry data were added.

L201 The assembly comprised 6491 would read better.
Corrected.

L202 73 splice variants seems remarkably few, in fact so few that it is questionable whether these are worth detailing and including as this simply highlights that this analysis is not at all

comprehensive.
We know, these are only few, but we feel that our data are reliable.

L225 This section is really weak. To make any such inference a proper analysis to identify signatures of selection using population resequencing data would be needed. The conclusions stated on the basis of heterozygous sites within a single individual have effectively no value and offer no real insight.
Even though we somewhat disagree, we shortened this section and tuned down some statements.

L240 Blasting is not a term. You mean sequence homology searches performed using BLAST. The same error is repeated at L243
Corrected.

L243 Correct 'eight out them'
Corrected.

L249 Detection, not disturbance
Corrected.

L257 provided, not provide
Corrected.

L258 There are actually quite a few tree genomes available now. I would actually argue that until the genome is annotated more comprehensively and the assembly improved, it is actually quite unlikely that this genome will be included in comparative studies.
See above comment. The genome is an integral part of an international beech population genomics effort. In addition, it should be noted that our assembly (and annotation) is comparable to highest quality tree genomes available.

Reviewer #3: Dear Authors

I want to congratulate you on the manuscript detailing the assembly of the European Beech. The manuscript is written in a clear and concise manner, and was a pleasure to review.

I would like to recommend the following corrections made to the manuscript prior to the publication. I refer to the manuscript numbers, not the page numbers:

Line:

58: "nature" should be "natural"
Corrected.

59: "roots is highly" should be "roots is also highly"
Corrected.

65: "debated" should be "debatable"
Corrected.

Table 1 should include the statistics for Eucalyptus (angiosperm)

Included.

Figure 2B: "Prop hetoerzygous sites" should be "Probability of heterozygous sites"
Prop. Stands for Proportion, outlined in the legend. We are sorry for the confusability.

Close
