# Supplementary material for: A reference genome of the European beech (Fagus sylvatica L.)
Source: Gigascience. 2018 May 28;7(6):giy063. doi: 10.1093/gigascience/giy063 (PMC6014182; doi:10.1093/gigascience/giy063)
Supplement: Reviewer_2_Report_(Original_Submission) -- Nathaniel Street — 2/19/2018 Reviewed [file giy063_reviewer_2_report_(original_submission).pdf]

Reviewer Report

Title: A reference genome of the European Beech (Fagus sylvatica L.)

Version: Original Submission **Date:** 2/19/2018

Reviewer name: Nathaniel Street

Reviewer Comments to Author:

Mishra et al. present the draft assembly of European beech. A very superficial and dry analysis is reported of basic assembly features. There is no repeat annotation, no assembly correctness assessment and a relatively unusual approach to gene annotation that presents potential users with two highly contrasting gene annotations that have not been merged or compared. For example, the BREAKER analysis identified almost twice as many genes as the BLAST2GO analysis - what are all those extra genes? Very little use is made of the RNA-Seq data, which is extremely limited. There is no presented analysis of how many genes were supported by RNA-Seq evidence and no way of ascertaining what, if anything, this single RNA-Seq sample contributed. Why were no analyses of gene families presented, for example to look for expanded gene families involved in fungal interactions? The presented results lack any biological insight or analysis and the detailed assembly characteristics are of limited to no interest.The authors should carefully check the manuscript, particularly the use of commas. There are a number of cases where a closing comma is required, making some sentences hard to read. However, the manuscript is generally clear and concise.As there is nothing novel, new or different to the DNA extraction employed for this work I suggest that reference to this be removed from the abstract.Although the annotation and analysis of the presented assembly are far from comprehensive, I see no obvious errors or problems with the described methodology. However, some further exploration of assembly quality at each step of the assembly would have been very useful for informing potential users as to the reliability of the assembly. There are a number of tools for performing such analyses using alignments of paired-end and jumping libraries. I would very much have liked to see this as it is far from clear whether the presented hybrid approach to combine the Illumina short read and PacBio read data was optimal and how successful this was. The authors do not state any justification for the selected methodology or indicate whether other options were explored. Was the combination of tools used an effectively ad hoc approach or were these informed choices?I confirm that the web resource linked to is functional, although it is of limited use and functionality.Abstract:Is the species important because it is a climax species in natural forests, because of its high value in planted stands or both? Mb should be Mb pans similarly for all Base Pair units stated throughout. In it a little odd to use BUSCO as if it is a common-use term in the abstract. It would be better in the abstract to say a set of benchmark eukaryotic conserved genes or similar. The conclusions section of the abstract is widely speculative, especially as there are no actual biological analyses presented in the study to support any of these claims.Keywords: It seems strange to list biodiversity and climate change as keywords for the sequencing of a single individualWhy are two citations styles used simultaneously?L65 This often-stated need for genomics data is a stretch. How will this genome sequence provide clear and immediate evidence about whether this species will cope with future climate conditions? Such tenuous justifications for the work are really not needed.L92 The authors claim to present a method for extracting contaminant-free DNA. What they actually did was to sample a dormant tissue that happens to have low microbiome abundance. There is nothing novel or unusual about this as a method. It would be far more appropriate to simply state that a tissue type with low abundance of bacteria and fungi was used for the DNA extraction.L93 Define CTAB and similarly always define abbreviations at first use.L95 When were the buds sampled?L117 It seems rather a strange choice to extract RNA to support gene annotation using only a dormant tissue type.L135 Here, and throughout, please state the versions of software used and all relevant parameters, stating default where appropriate.L144 How was this k-mer length selected? It is relatively high. What is the expected heterozygosity of beech as this interacts with k-mer length to affect assembly outcome. I also do not understand using a long k-mer here and then a much shorter k-mer length for the hybrid assembly.L157 The gene annotation approach is rather unusual. Why were no ab initio or evidence-based annotation

pipelines applied? The annotation as presented does not appear to be particularly comprehensive and would miss genes not expressed or not represented in the undefined Arabidopsis dataset.L159 Were the intron size settings for TopHat2 adjusted to reflect plant species?L160 What is a pre-trained dataset?L162 What does 'Otherwise default values were opted' mean? What is this referring to? L172 I find the described methodology for locating heterozygous positions hard to follow. Were SNPs called using the reads alignments or did this reply only on called sites from the assembly? It is far more common to align reads and to then use a tool such as GATK to call heterozygous positions.L187 It is not clear what a BLAST search against Fungi means here? What is the input, exactly, to construct the BLAST index used for this sequence homology search? I also do not understand the logic and why this search was not directly performed to the NCBI NR database.L200 k-mer based genome size estimates can be very inaccurate. Are there any flow-cytometry measures available?L201 The assembly comprised 6491 would read better.L202 73 splice variants seems remarkably few, in fact so few that it is questionable whether these are worth detailing and including as this simply highlights that this analysis is not at all comprehensive.L225 This section is really weak. To make any such inference a proper analysis to identify signatures of selection using population resequencing data would be needed. The conclusions stated on the basis of heterozygous sites within a single individual have effectively no value and offer no real insight.L240 Blasting is not a term. You mean sequence homology searches performed using BLAST. The same error is repeated at L243L243 Correct 'eight out them'L249 Detection, not disturbanceL257 provided, not provideL258 There are actually quite a few tree genomes available now. I would actually argue that until the genome is annotated more comprehensively and the assembly improved, it is actually quite unlikely that this genome will be included in comparative studies.

Level of Interest

Please indicate how interesting you found the manuscript: An article whose findings are important to those with closely related research interests

Quality of Written English

Please indicate the quality of language in the manuscript: Acceptable

Declaration of Competing Interests

Please complete a declaration of competing interests, considering the following questions:

- Have you in the past five years received reimbursements, fees, funding, or salary from an organisation that may in any way gain or lose financially from the publication of this manuscript, either now or in the future?
- Do you hold any stocks or shares in an organisation that may in any way gain or lose financially from the publication of this manuscript, either now or in the future?
- Do you hold or are you currently applying for any patents relating to the content of the manuscript?
- Have you received reimbursements, fees, funding, or salary from an organization that holds or has applied for patents relating to the content of the manuscript?
- Do you have any other financial competing interests?

- Do you have any non-financial competing interests in relation to this paper?

If you can answer no to all of the above, write 'I declare that I have no competing interests' below. If your reply is yes to any, please give details below.

I declare that I have no competing interests

I agree to the open peer review policy of the journal. I understand that my name will be included on my report to the authors and, if the manuscript is accepted for publication, my named report including any attachments I upload will be posted on the website along with the authors' responses. I agree for my report to be made available under an Open Access Creative Commons CC-BY license (http://creativecommons.org/licenses/by/4.0/). I understand that any comments which I do not wish to be included in my named report can be included as confidential comments to the editors, which will not be published.

I agree to the open peer review policy of the journal

To further support our reviewers, we have joined with Publons, where you can gain additional credit to further highlight your hard work (see: https://publons.com/journal/530/gigascience). On publication of this paper, your review will be automatically added to Publons, you can then choose whether or not to claim your Publons credit. I understand this statement.

Yes
